# Functional Role of Lanthanides in Enzymatic Activity and Transcriptional Regulation of Pyrroloquinoline Quinone-Dependent Alcohol Dehydrogenases in *Pseudomonas putida* KT2440

**DOI:** 10.1128/mBio.00570-17

**Published:** 2017-06-27

**Authors:** Matthias Wehrmann, Patrick Billard, Audrey Martin-Meriadec, Asfaw Zegeye, Janosch Klebensberger

**Affiliations:** a University of Stuttgart, Institute of Technical Biochemistry, Stuttgart, Germany; b Université de Lorraine, LIEC UMR7360, Faculté des Sciences et Technologies, Vandoeuvre-lès-Nancy, France; c CNRS, LIEC UMR7360, Faculté des Sciences et Technologies, Vandoeuvre-lès-Nancy, France; California Institute of Technology (currently at Princeton University); California Institute of Technology/HHMI

**Keywords:** pyrroloquinoline quinone, *Pseudomonas putida*, alcohol dehydrogenase, functional redundancy, gene regulation, lanthanides, protein function, volatiles

## Abstract

The oxidation of alcohols and aldehydes is crucial for detoxification and efficient catabolism of various volatile organic compounds (VOCs). Thus, many Gram-negative bacteria have evolved periplasmic oxidation systems based on pyrroloquinoline quinone-dependent alcohol dehydrogenases (PQQ-ADHs) that are often functionally redundant. Here we report the first description and characterization of a lanthanide-dependent PQQ-ADH (PedH) in a nonmethylotrophic bacterium based on the use of purified enzymes from the soil-dwelling model organism *Pseudomonas putida* KT2440. PedH (PP_2679) exhibits enzyme activity on a range of substrates similar to that of its Ca^2+^-dependent counterpart PedE (PP_2674), including linear and aromatic primary and secondary alcohols, as well as aldehydes, but only in the presence of lanthanide ions, including La^3+^, Ce^3+^, Pr^3+^, Sm^3+^, or Nd^3+^. Reporter assays revealed that PedH not only has a catalytic function but is also involved in the transcriptional regulation of *pedE* and *pedH*, most likely acting as a sensory module. Notably, the underlying regulatory network is responsive to as little as 1 to 10 nM lanthanum, a concentration assumed to be of ecological relevance. The present study further demonstrates that the PQQ-dependent oxidation system is crucial for efficient growth with a variety of volatile alcohols. From these results, we conclude that functional redundancy and inverse regulation of PedE and PedH represent an adaptive strategy of *P. putida* KT2440 to optimize growth with volatile alcohols in response to the availability of different lanthanides.

## IMPORTANCE

Because of their low bioavailability, lanthanides have long been considered biologically inert. In recent years, however, the identification of lanthanides as a cofactor in methylotrophic bacteria has attracted tremendous interest among various biological fields. The present study reveals that one of the two PQQ-ADHs produced by the model organism *P. putida* KT2440 also utilizes lanthanides as a cofactor, thus expanding the scope of lanthanide-employing bacteria beyond the methylotrophs. Similar to the system described in methylotrophic bacteria, a complex regulatory network is involved in lanthanide-responsive switching between the two PQQ-ADHs encoded by *P. putida* KT2440. We further show that the functional production of at least one of the enzymes is crucial for efficient growth with several volatile alcohols. Overall, our study provides a novel understanding of the redundancy of PQQ-ADHs observed in many organisms and further highlights the importance of lanthanides for bacterial metabolism, particularly in soil environments.

## INTRODUCTION

As a soil-dwelling organism, *Pseudomonas putida* can encounter a large diversity of volatile organic compounds (VOCs) from different sources (1–3). The ecological role of many VOCs is not clearly defined, but the number of known specific functions is rapidly increasing. These functions include the growth promotion of plants; antiherbivore, antibacterial, and antifungal activities; and signaling both within the same species and between different species (4–7). Among many other chemicals, VOCs include cyclic, acyclic, aromatic, and terpenoid structures with alcohol and aldehyde moieties, which are derived mainly from the metabolism of bacterial, yeast, fungal, or plant species. Beside their specific molecular function, they can also serve as carbon and energy sources for a wide range of microorganisms. To use volatile alcohols and aldehydes efficiently, it is advantageous if their metabolism is initiated by pyrroloquinoline quinone-dependent alcohol dehydrogenases (PQQ-ADHs), for at least two different reasons. First, by using a periplasmic oxidation system, the organism is able to rapidly detoxify the often harmful chemicals without prior transport into the cytoplasm (8, 9). Second, the periplasmic location of the enzymes allows the rapid capture of a volatile carbon source by the conversion to and accumulation of acidic products with decreased volatility.

The reaction mechanism of PQQ-ADHs is still not completely resolved but most likely proceeds via an ion-assisted direct hydride transfer from the substrate to C-5 of the noncovalently bound cofactor PQQ (10–12). PQQ-ADHs can be divided into different subclasses, depending either on their molecular composition (quinoproteins or quinohemoproteins) or whether they are membrane bound or freely soluble within the periplasm (13). Many organisms express different classes or even multiple PQQ-ADHs of the same type, indicating the importance of these enzymes (14–16). The genome of *P. putida* KT2440 encodes two PQQ-ADHs, namely, PP_2674 (*pedE*) and PP_2679 (*pedH*), which have been shown to be involved in the metabolism of different substrates (17–19). PedE is a homolog of ExaA from *Pseudomonas aeruginosa*, which is the most intensively studied member of the class of soluble ethanol dehydrogenases (20–24). ExaA and homologs thereof accept a wide variety of substrates and rely on a Ca^2+^ ion in the active site, in addition to the PQQ cofactor, for the oxidation of primary and secondary alcohols, as well as aldehydes (18, 24). Despite their broad substrate range, ExaA-like enzymes show only very poor conversion of methanol. Not surprisingly, methano- and methylotrophic bacteria, which can use methane and methanol as a source of carbon and energy, encode a different type of PQQ-dependent enzyme, the MxaF-type of methanol dehydrogenase (MxaF-MDH) (25, 26). These enzymes display high substrate specificity for methanol and formaldehyde and also depend on Ca^2+^ as a cofactor (27). Interestingly, methano- and methylotrophic bacteria encode an additional type of PQQ-dependent MDH, the XoxF-type, which utilizes rare earth metals (REMs) of the lanthanide series as cofactors instead of calcium (28–30).

Since their discovery, several XoxF-type MDHs from different methano- and methylotrophs have been identified and characterized (31–33). Phylogenetic analysis of available sequence information suggests that lanthanide dependency is an ancestral feature of PQQ-ADHs and that these enzymes are more abundant than their Ca^2+^-dependent counterparts (15, 34). In addition, a very recent publication described the first lanthanide-dependent ethanol dehydrogenase in *Methylobacterium extorquens* AM1 (16). As a consequence, REM-dependent enzymes and the microorganisms that produce them have sparked a lot of academic and commercial interest, as they might be exploited in a broad variety of biotechnological fields (35, 36). Potential applications range from the development of new biocatalysts and biosensors to the use of the associated microorganisms in REM biomining, bioleaching, and recycling processes. However, so far, lanthanide-dependent PQQ-ADHs have been limited to methano- and methylotrophic bacteria.

Here, we offer the first description and detailed characterization of a lanthanide-dependent PQQ-ADH (PedH) in the nonmethylotrophic bacterium *P. putida* KT2440, which is a model organism for industrial and environmental applications (37–40). We demonstrate that PedH exhibits enzymatic activity only in the presence of lanthanides, including, but not limited to, lanthanum, praseodymium, and cerium, and show that this enzyme has a substrate range similar to that of PedE, the recently characterized Ca^2+^-dependent PQQ-ADH from KT2440 (18). By the use of deletion mutants and transcriptional reporter fusions, we provide evidence that the functional redundancy of the PQQ-ADHs reflects the variable availability of lanthanides in the natural environment of *P. putida* KT2440 and show that these enzymes are crucial for efficient growth with a variety of volatile alcohols. Finally, we reveal that PedH plays an important role in the regulatory switch between the transcription of *pedH* and that of *pedE*, most likely acting as a sensory module. From these data, we conclude that KT2440 responds to lanthanide availability with the inverse transcriptional regulation of the two PQQ-ADHs to optimize growth with volatile alcoholic and aldehyde substrates.

## RESULTS

### Biochemical characterization of PedE and PedH.

Like many other organisms, *P. putida* KT2440 harbors more than one gene annotated as a PQQ-ADH, namely, PP_2674 (PedE/QedH; GenInfo Identifier [GI]: 26989393) and PP_2679 (PedH; GI: 26989398). To study the rationale for this redundancy, we purified and characterized the corresponding enzymes. A one-step affinity chromatography method produced soluble C-terminally His-tagged PedE and PedH to visible purity (see Fig. S1 in the supplemental material) from cell lysates of *Escherichia coli* BL21(DE3). Under optimized reaction conditions, which include the presence of 1 mM Ca^2+^, the specific activities of purified PedE with a variety of substrates were determined (Table 1). For all linear primary alcohols and aldehydes, comparably high enzyme activities ranging from 1.9 ± 0.2 to 6.7 ± 0.9 U mg^−1^ were found. Similarly, 2-phenylethanol, the secondary alcohol 2-butanol, cinnamyl alcohol, and the acyclic sesquiterpene farnesol were efficiently converted, with specific activities ranging from 6.7 ± 1.1 to 2.0 ± 0.3 U mg^−1^. Methanol, 2,3-butanediol, and ethanolamine were poor substrates for the enzyme, with about 10-fold lower specific activity than ethanol or 2-phenylethanol. Of all of the substrates tested, cinnamyl aldehyde was the only compound with which no PedE activity was detected.

10.1128/mBio.00570-17.2 FIG S1  SDS-PAGE analysis of PedE and PedH production. Ten microliters of cell extract or 20 µg of purified protein was loaded per lane. Download FIG S1, TIF file, 1.1 MB. Copyright © 2017 Wehrmann et al. 2017 Wehrmann et al. This content is distributed under the terms of the Creative Commons Attribution 4.0 International license.

**TABLE 1:** Specific activities of PedE and PedH with various alcohols and aldehydes

**Substrate**	**Mean specific activity (U mg**^**−1**^**) ± SD**^d^	
	**PedE**	**PedH**
Methanol	0.61 ± 0.10	0.80 ± 0.05
Ethanol	6.7 ± 0.9	11.0 ± 0.3
Ethanolamine	0.55 ± 0.09	1.6 ± 0.2
1-Butanol	5.8 ± 0.1	11.5 ± 0.7
2-Butanol	4.4 ± 0.7	7.6 ± 0.4
2,3-Butanediol	0.39 ± 0.03	0.78 ± 0.04
1-Hexanol	5.2 ± 0.1	10.4 ± 1.1
1-Octanol^a^	3.5 ± 0.2	4.7 ± 0.7
2-Phenylethanol	6.7 ± 1.1	10.2 ± 1.4
Acetaldehyde	4.7 ± 0.5	6.7 ± 0.4
Butyraldehyde	6.1 ± 0.4	10.3 ± 0.6
Hexanal^a^	3.8 ± 0.1	6.2 ± 0.3
Octanal^a^	1.9 ± 0.2	3.6 ± 0.6
Cinnamyl alcohol^a^	2.4 ± 0.1	3.9 ± 0.1
Cinnamaldehyde^b^	ND^c^	ND^c^
Farnesol^b^	2.0 ± 0.3	3.8 ± 0.5

^a^ Substrate concentration of 10 mM in DMSO.

^b^ Substrate concentration of 500 mM in DMSO.

^c^ ND, activity below detection limit.

^d^ Data are presented as the mean value of three independent measurements and the corresponding standard deviation. A substrate concentration of 10 mM in H_2_O was used if not indicated otherwise.

**FIG 1:**
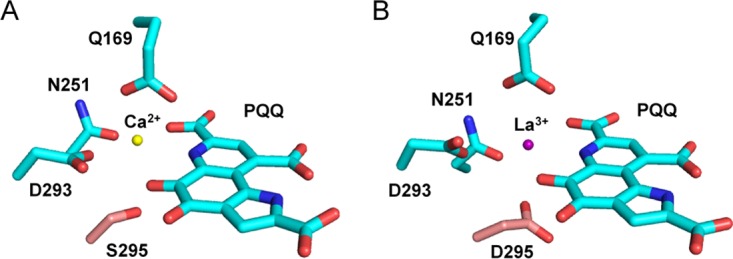
Homology models of PedE (A) (GI: 26989393) and PedH (B) (GI: 26989398) generated with SWISS-MODEL on the basis of the crystal structure of ExaA from *P. aeruginosa* (PDB code 1FLG) and visualized with PyMOL (70). The catalytic cation (yellow or violet sphere)-coordinating amino acids and the PQQ cofactor are shown as sticks with an element color code (C, cyan; O, red; N, blue). The amino acid at position 295 in PedE and PedH is highlighted by using a different color code (C, light red).

**FIG 2:**
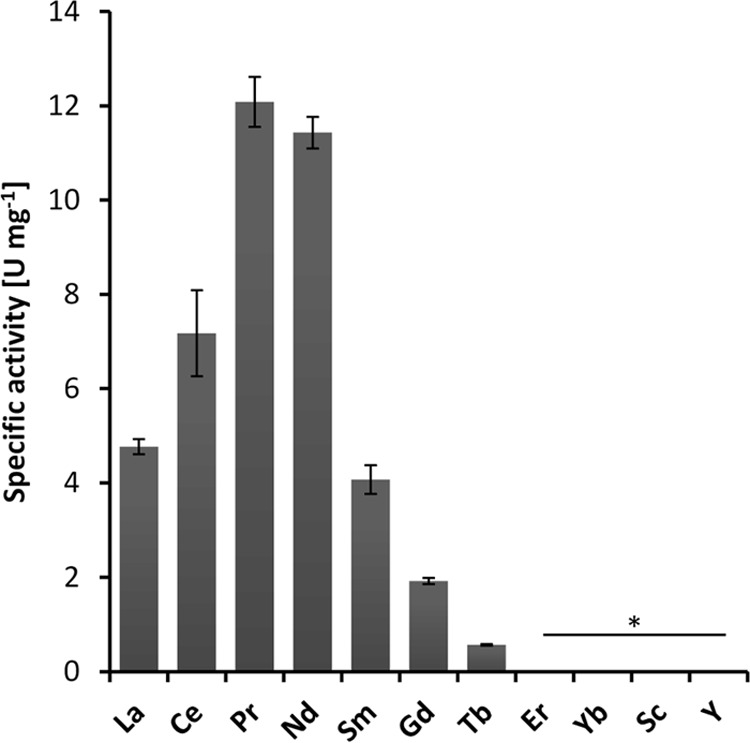
Specific activities of PedH in the presence of various REM ions at 1 µM with 10 mM ethanol as the substrate. Activities below the detection limit are indicated (*). Data are presented as the mean value of three replicates, and error bars represent the corresponding standard deviation.

When we assayed purified PedH under the optimized reaction conditions used for PedE, no activity was observed with any of the substrates tested (data not shown). Comparison of the active sites of both enzymes using homology models based on the crystal structure of the ethanol dehydrogenase ExaA of *P. aeruginosa* (PDB code 1FLG) revealed that, similar to other characterized representatives of the PQQ-dependent ethanol dehydrogenase type, the PedE protein harbors a serine residue at amino acid position 295 that is involved in the coordination of the Ca^2+^ ion (Fig. 1A). In contrast, in PedH, this residue is an aspartate (Fig. 1B). As this aspartate residue has recently been associated with the coordination of trivalent lanthanide ions in the active site of PQQ-dependent methanol and ethanol dehydrogenases in methylotrophs (15, 16), we tested PedH for activity with ethanol in the presence of a variety of REMs (Fig. 2). From these experiments we found that PedH showed no activity when 1 µM Er^3+^, Sc^3+^, Y^3+^, or Yb^3+^ was added to the reaction mixture. However, in the presence of the lanthanide La^3+^, Ce^3+^, Pr^3+^, Nd^3+^, Sm^3+^, Gd^3+^, or Tb^3+^ at 1 µM, enzymatic activity was detected, with maximal specific activity observed with Pr^3+^ and Nd^3+^ and only very low activity observed with Gd^3+^ and Tb^3+^.

**FIG 3:**
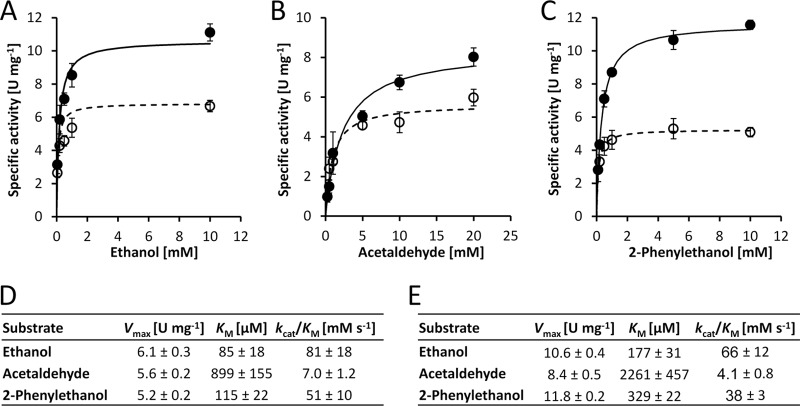
Kinetic parameter determination. (A to C) Michaelis-Menten plots showing the specific activities of the enzymes PedE (black circles) and PedH (white circles) in various concentrations of ethanol (A), acetaldehyde (B), and 2-phenylethanol (C). For PedE, 1 mM CaCl_2_ and 50 µM PQQ were used in the reaction mixture, while for PedH, 1 µM PrCl_3_ and 1 µM PQQ were used. The data are shown as the mean value of triplicate measurements with error bars representing the standard deviation. The maximum velocities (*V*_max_), substrate affinities (*K*_M_), and catalytic efficiencies (*k*_cat_/*K*_M_, were *k*_cat_ is the turnover frequency per cofactor molecule) of PedE (D) and PedH (E) for different substrates were derived from panels A to C by nonlinear regression to a Michaelis-Menten model (continuous lines for PedH and dashed lines for PedE). Kinetic constants are presented as best-fit values ± the standard errors.

Under optimized conditions, which included supplementation with 1 µM Pr^3+^ instead of Ca^2+^, PedH showed an activity pattern similar to that of PedE (Table 1) but exhibited about 2-fold higher specific activity. Further, the functional concentration range of the metal cofactor differed dramatically for the two enzymes (Fig. S2A). While PedE showed enzyme activity at concentrations of 10 µM to 10 mM CaCl_2_, with a peak in activity at 1 mM, PedH activity was found with lanthanide concentrations as low as 10 nM and up to 100 µM, with a peak in activity at 1 µM. From these data, we calculated the dissociation constant (*K*_*d*_) for various metals and the corresponding enzymes and found that PedH has an 850- to 2,500-fold higher binding affinity for lanthanides (*K*_*d*_ = 25 to 75 nM; Fig. S2B2) than PedE does for Ca^2+^ (*K*_*d*_ = 64 µM; Fig. S2B1). The subsequent determination of kinetic parameters with ethanol showed that the maximal velocity (*V*_max_) of PedH was approximately 1.7-fold higher than that of PedE (10.6 versus 6.1 U mg^−1^; Fig. 3). However, the corresponding binding constant (*K*_M_) of PedE was 2-fold lower than that of PedH (85 versus 177 µM). A similar pattern was found with acetaldehyde and 2-phenylethanol, but with catalytic efficiencies approximately 1.6-fold (2-phenylethanol) and 10- to 15-fold (acetaldehyde) lower than those measured with ethanol. Statistical analysis (two-tailed *t* test; α = 0.05; *n* = 3; GraphPad Prism version 7.03) revealed that all *V*_max_ and *K*_M_ values, except for the *K*_M_ with ethanol, were significantly different (*P* < 0.05) between PedE and PedH; however, no significant difference in catalytic efficiency (*k*_cat_/*K*_M_ ratio) was observed.

10.1128/mBio.00570-17.3 FIG S2  (A) Specific activities of PedE (dashed line) or PedH (solid lines) in the presence of different concentrations of Ca^2+^ (empty cycles), La^3+^ (dark circles), Ce^3+^ (black diamonds), or Pr^3+^ (black squares) ions. (B) Specific activities of PedE (B1) or PedH (B2) at different metal concentrations. Data were fitted to a one-site binding model by least-squares analysis, and affinity constants of PedE for Ca^2+^ (K_*d*_ = 64 µM) and PedH for Pr^3+^ (*K*_*d*_ = 75 nM), Ce^3+^ (K_*d*_ = 25 nM), and La^3+^ (K_*d*_ = 30 nM) were calculated. Download FIG S2, TIF file, 2.4 MB. Copyright © 2017 Wehrmann et al. 2017 Wehrmann et al. This content is distributed under the terms of the Creative Commons Attribution 4.0 International license.

### Growth with volatile alcohols in the presence or absence of lanthanides.

**FIG 4:**
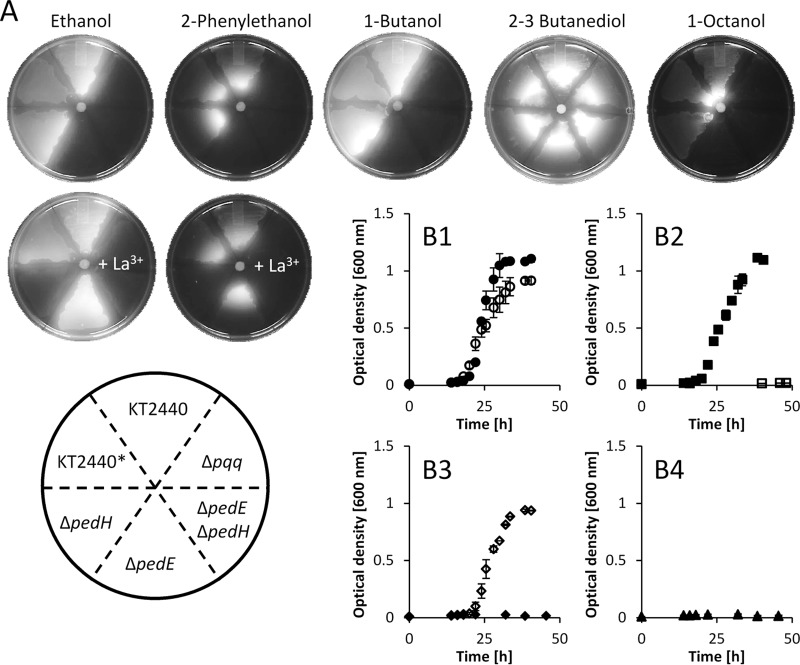
(A) Growth with various substrates (10-µl drop of a 1:1 mixture with DMSO) on M9 agar plates. Growth was quantified with a digital imaging system after 48 h with combined white light and UV illumination (excitation wavelength. 254 nm). All pictures were sized, isolated from the background, and corrected for sharpness (+50%), brightness (+20%), and contrast (+40%). (B1 to B4) growth of KT2440* (Δ*upp* strain used as the parental strain for knockout mutants; B1, circles), a Δ*pedE* strain (B2, diamonds), a Δ*pedH* strain (B3, squares), and a Δ*pedE* Δ*pedH* strain (B4, triangles) in M9 medium with 5 mM 2-phenylethanol in the absence (black symbols) or presence (open symbols) of 20 µM La^3+^. Bacteria were grown in 25 ml (125-ml plastic Erlenmeyer flasks) at 30°C with shaking at 180 rpm (Multifors), and growth was quantified by OD_600_ measurements. Data represent the mean of two individual cultures with error bars representing the corresponding range. All error bars are depicted but might not be visible because of the size of the corresponding symbol used for the mean value.

In a next step, individual (Δ*pedE*, Δ*pedH*, and Δ*pqq*) and combinatorial (Δ*pedE* Δ*pedH*) deletion mutants were tested for growth with several VOCs in an agar plate assay in the presence or absence of 20 µM lanthanum (Fig. 4A). Strains KT2440 (type strain) and KT2440* (Δ*upp* strain used as the parental strain for knockout mutants) and a Δ*pedH* mutant grew efficiently with ethanol, 1-butanol, and 2-phenylethanol in the absence of La^3+^. A Δ*pedE* strain displayed no growth under this condition. Even more interestingly, the addition of 20 µM La^3+^ to the agar medium not only resulted in growth of the Δ*pedE* strain but also restricted the growth of the Δ*pedH* strain. The Δ*pedE* Δ*pedH* double mutant and the Δ*pqq* mutant, which is deficient in PQQ biosynthesis, showed no growth under both conditions. These experiments revealed that efficient growth with all of the alcohols tested, except the microbial fermentation product 2,3-butanediol, was dependent on the functional expression of PedE or PedH.

To validate these findings, experiments testing growth in liquid M9 medium with 2-phenylethanol as the sole carbon and energy source in the presence or absence of 20 µM La^3+^ were performed (Fig. 4B1 to B4). Plastic Erlenmeyer flasks were used to avoid potential contaminations of REMs from the glassware (33). Growth of the liquid cultures followed a pattern similar to that observed in the agar plate assay. While strain KT2440* (Fig. 4B1) showed growth after an 18- to 20-h lag phase and a peak in optical density (OD) at about 35 h under both conditions, the absence and presence of lanthanum, the Δ*pedE* Δ*pedH* double mutant (Fig. 4B4) did not display growth under either condition. Growth of the Δ*pedE* strain (Fig. 4B3) was observed exclusively in the presence of lanthanum. Lastly, the Δ*pedH* strain (Fig. 4B2) showed growth similar to that of strain KT2440* in the absence of lanthanum, but no growth was detected when 20 µM La^3+^ was added.

### Transcriptional regulation of *pedE* and *pedH* determines growth with alcoholic volatiles.

**FIG 5:**
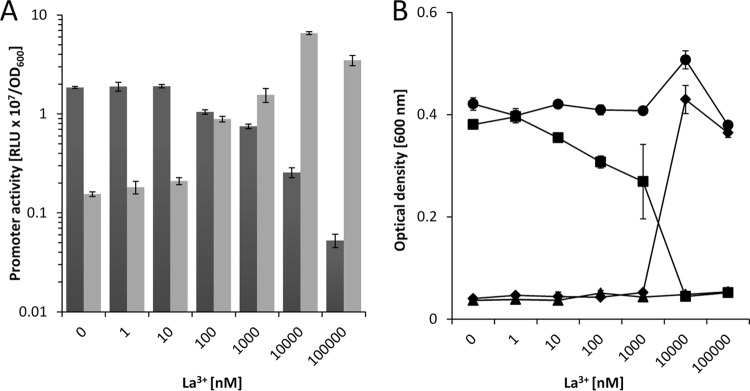
(A) Activities of the *pedE* (dark gray bars) and *pedH* (light gray bars) promoters in strain KT2440* during incubation in liquid M9 medium supplemented with 1 mM 2-phenylethanol in the presence of various concentrations of La^3+^. Promoter activities are in relative light units (RLU × 10^7^) normalized to the OD_600_. (B) The growth of KT2440* (black circles), a Δ*pedE* strain (black diamonds), a Δ*pedH* strain (black squares), and a Δ*pedE* Δ*pedH* strain (black triangles) in liquid M9 medium with 5 mM 2-phenylethanol in the presence of different concentrations of La^3+^ was determined as the OD_600_ after incubation at 30°C for 24 h. Data are presented as mean values of biological triplicates, and error bars represent the corresponding standard deviations.

The previous experiments proved that for efficient growth with various VOCs, the functional expression of at least one of the PQQ-ADHs is essential. The growth inhibition of the Δ*pedH* strain in the presence of lanthanum indicated a potential repression of the *pedE* gene in the presence of lanthanides, similar to recent reports on different methylotrophic bacteria (41–43). To test this hypothesis, we constructed two reporter strains suitable for probing *pedE* and *pedH* promoter activities in KT2440*. When these strains were tested with 1 mM 2-phenylethanol in M9 medium (Fig. 5A), the addition of up to 10 nM La^3+^ did not affect *pedE* promoter activity compared to that measured in the absence of lanthanum. In contrast, the presence of 100 nM to 100 µM La^3+^ resulted in reduced *pedE* promoter activity. An inverse pattern was found for the *pedH* promoter. Here, very low activities were detected in the presence of up to 10 nM La^3+^. Upon the addition of lanthanum at ≥100 nM, expression from the *pedE* promoter was induced, with a peak at 10 µM.

The importance of the transcriptional regulation of *pedE* and *pedH* was further tested by growth experiments with 2-phenylethanol in liquid M9 medium (Fig. 5B). Growth of KT2440* was not affected by the addition of up to 100 µM lanthanum. On the other hand, the Δ*pedH* strain showed a linear decrease in growth in the presence of increasing La^3+^ concentrations up to 1 µM and no measurable growth when La^3+^ was present in the medium at ≥10 µM. In contrast, growth of the Δ*pedE* strain was observed only in the presence of La^3+^ at ≥10 µM. The Δ*pedE* Δ*pedH* strain did not grow under any of the conditions tested. A similar correlation between growth and *pedE* and *pedH* promoter activity was observed with Ce^3+^, Pr^3+^, Nd^3+^, and Sm^3+^ (Fig. S3).

10.1128/mBio.00570-17.4 FIG S3  (A) Growth of Δ*pedE* (light gray bars) and Δ*pedH* (dark gray bars) strains after incubation for 24 h at 30°C in liquid M9 minimal medium containing 5 mM 2-phenylethanol as a source of carbon and energy supplemented with different REMs at 20 µM. (B) Activities of the *pedE* (dark gray bars) and *pedH* (light gray bars) promoters in strain KT2440* in liquid MP minimal medium containing 1 mM 2-phenylethanol supplemented with different REMs at 20 µM. Promoter activities are presented in relative light units (RLU) normalized to the OD_600_. Bars represent the mean of three biological replicates, and error bars show the corresponding standard deviation. Download FIG S3, TIF file, 2.3 MB. Copyright © 2017 Wehrmann et al. 2017 Wehrmann et al. This content is distributed under the terms of the Creative Commons Attribution 4.0 International license.

**FIG 6:**
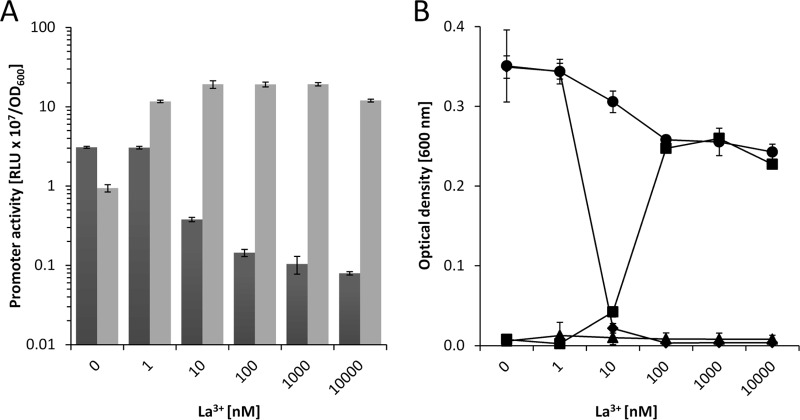
(A) Activities of the *pedE* (dark gray bars) and *pedH* (light gray bars) promoters in strain KT2440* during incubation in liquid MP medium supplemented with 1 mM 2-phenylethanol in the presence of various concentrations of La^3+^. Promoter activities are in relative light units (RLU × 10^7^) normalized to the OD_600_. (B) The growth of KT2440* (black circles), a Δ*pedE* strain (black diamonds), a Δ*pedH* strain (black squares), and a Δ*pedE* Δ*pedH* strain (black triangles) in liquid MP medium with 5 mM 2-phenylethanol in the presence of different La^3+^ concentrations was determined as the OD_600_ after incubation at 30°C for 24 h. Data are presented as the mean values of biological triplicates, and error bars represent the corresponding standard deviations.

These results demonstrate that KT2440 inversely regulates *pedE* and *pedH* promoter activity in response to various lanthanide concentrations and suggests that this regulation is the primary determinant of growth. However, in comparison with earlier studies with *M. extorquens* AM1, the effective lanthanide concentration needed for growth was much higher (10 µM versus 5 nM) (41). As lanthanides are known to form very poorly soluble complexes with phosphate and hydroxide ions, we wondered whether this difference was caused by the minimal medium used for growth (MP versus M9). When the experiments were repeated with MP medium, the same general trend and correlation of promoter activity and growth were found that were described for M9 medium (Fig. 6A and B). However, one difference was that the effective lanthanum concentrations needed to trigger a transcriptional response and growth were considerably lower (1 and 10 nM). Another difference was that at a concentration of 10 nM La^3+^, minimal growth of both single mutant strains was observed. The latter observation indicated that environmental conditions might exist under which PedE and PedH are both functionally produced. To further test this hypothesis, an additional growth experiment was performed that indeed showed growth of both single mutants in a concentration range of 1 to 15 nM La^3+^ after prolonged (48 h) incubation (Fig. S9).

### Impact of PedE and PedH on transcriptional regulation.

**FIG 7:**
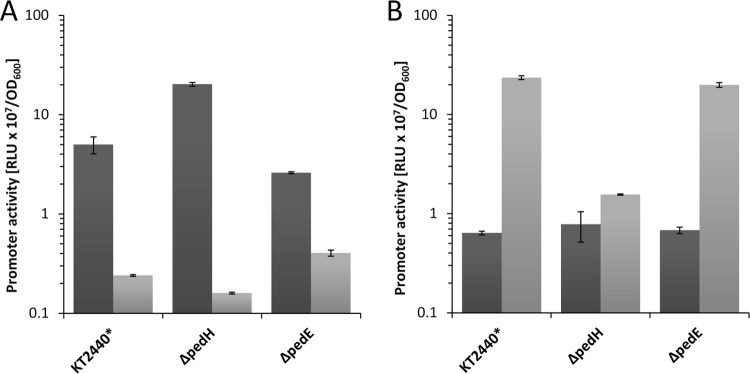
Activities of the *pedE* (A) and *pedH* (B) promoters in strain KT2440* (Δ*upp* strain used as the parental strain for knockout mutants), a Δ*pedE* strain, and a Δ*pedH* strain in liquid MP medium with 1 mM 2-phenylethanol in the absence (dark gray bars) or presence (light gray bars) of 10 nM La^3+^. Promoter activities are in relative light units (RLU × 10^7^) normalized to the OD_600_. Data are presented as the mean values of biological triplicates, and error bars represent the corresponding standard deviations. The promoter activities of each strain in the presence or absence of lanthanides were statistically analyzed (two-tailed *t* test; α = 0.05; *n* = 3; GraphPad Prism version 7.03) and found to be significantly different (*P* < 0.01).

In *M. extorquens* AM1, the transcription of MDHs is regulated, at least partially, by the PQQ-dependent enzymes themselves (44). To test whether similar outside-in signaling is also present in *P. putida* KT2440, expression from the *pedE* and *pedH* promoters was quantified during growth with 2-phenylethanol in MP medium (Fig. 7). In the absence of lanthanum, the Δ*pedH* strain showed a 4-fold induction of *pedE* promoter activity, whereas the Δ*pedE* strain exhibited a slight (0.5-fold) decrease in expression from the *pedE* promoter compared to that of KT2440* (Fig. 7A). The presence of 10 nM La^3+^ resulted in a strong reduction of *pedE* promoter activity in all of the strains tested (21-fold for KT2440*, 6-fold for the Δ*pedE* strain, 127-fold for the Δ*pedH* strain) compared to the control without lanthanum. In comparison with expression from the *pedE* promoter, expression from the *pedH* promoter was considerably lower for all of the strains in the absence of lanthanum (Fig. 7B). However, when lanthanum was present, strong induction of *pedH* promoter activity in strain KT2440* (37-fold) and the Δ*pedE* strain (29-fold) was detected. Notably, the expression from the *pedH* promoter was dramatically reduced (2-fold versus 37-fold) in the Δ*pedH* strain in comparison with that in the strains capable of producing a functional PedH protein.

## DISCUSSION

Lanthanide-dependent enzymes have so far been found exclusively within methylotrophic organisms (16, 28–30, 32, 33). Using purified enzymes, we discovered that PedH, one of the two PQQ-ADHs produced by the nonmethylotrophic model organism *P. putida* KT2440, is also a lanthanide-dependent enzyme that utilizes La^3+^, Ce^3+^, Pr^3+^, Nd^3+^, Sm^3+^, Gd^3+^, and Tb^3+^ as metal cofactors. The highest catalytic rates were observed with Pr^3+^ and Nd^3+^. Notably, with lanthanides with atomic masses higher than that of Nd^3+^, the specific activity decreased gradually, eventually resulting in no detectable activity with the heaviest lanthanides tested (Er^3+^ and Yb^3+^). An analogous effect was previously reported by Pol et al., who investigated the impact of different lanthanides on the growth rate of *Methylacidiphilum fumariolicum* SolV (33). A possible explanation for these observations is that the decreased atomic radius, which is a consequence of lanthanide contraction, keeps the heavier lanthanides from being functionally incorporated into the active site of PedH (45).

Kinetic parameters determined with Pr^3+^ and the three model substrates ethanol, acetaldehyde, and 2-phenylethanol revealed that the *V*_max_ of PedH is about 2-fold higher than that of its Ca^2+^-dependent counterpart PedE. It has been proposed that the increased activity can be explained by the higher Lewis acidity of the lanthanides compared to calcium (46). Interestingly, we found that the *K*_M_ values of the lanthanide-dependent enzyme PedH with acetaldehyde or 2-phenylethanol are significantly lower than those of the Ca^2+^-dependent protein PedE. One possible explanation for this result is the higher polarity that arises in the active pocket of the trivalent cation coordinating PedH compared to the divalent cation coordinating PedE. Another explanation could be a smaller catalytic pocket because of the larger atomic radius of Pr^3+^ than of Ca^2+^. The latter argument was proposed in an earlier report of a study with the MxaF-type MDH of *M. extorquens* AM1 as a reason for decreased activities when Ca^2+^ was replaced with Ba^2+^ (47). In any case, apart from the approximately 2-fold higher specific activity of PedH compared to PedE, the substrate scopes and catalytic efficiencies (*k*_cat_/*K*_M_) of both enzymes were found to be similar, which suggests that both enzymes are functionally redundant and differ only in their cofactor dependency.

Functional redundancy is a well-known mechanism to improve robustness in complex systems (48). The fact that many organisms express multiple PQQ-ADHs can be interpreted as an adaptation to maintain an important function under variable environmental conditions or in different microhabitats. Our study is supportive of such a hypothesis, as we demonstrate that under conditions of high lanthanide availability, efficient growth of cells with various naturally occurring alcoholic VOCs relies on the functional production of the lanthanide-dependent ADH PedH. Similarly, for growth in the absence of lanthanides, functional production of the calcium-dependent ADH PedE is mandatory. In this context, it is important to note that growth in the agar plate assay used in this study is restrictive, as it depends on diffusion and evaporation of the volatile substrates. Thus, the assay most likely cannot discriminate between substrates for which PedE or PedH function is essential and substrates for which other but less efficient catabolic routes exist. We found that growth on ethanol (unpublished data) and 1-butanol (19), but not growth on 2-phenylethanol (this study), is possible but less efficient for a strain lacking PedE and PedH. From our data and data from a previously published study (49), we therefore conclude that beside the fact that PedE and PedH are not essential for growth with short-chain (C_2_ to C_5_) aliphatic alcohols, both enzymes provide rapid conversion of these substrates, which is crucial for efficient growth under restrictive conditions.

We further show that growth phenotypes strongly correlate with inverse transcriptional regulation of *pedE* and *pedH*. Similar results have been reported for several methylotrophic bacteria (41, 42). When cells of *P. putida* KT2440 were grown in liquid MP medium, the addition of as little as 10 nM lanthanides was sufficient to trigger *pedE* repression and a strong (20-fold) concomitant induction of *pedH*. The transcription of *pedH* was found to be strongly influenced by the PedH protein itself, implying a role for PedH as a lanthanide sensory module. In *M. extorquens* AM1, the transcription of the calcium-dependent MDH *mxaF* strictly relies on the presence of XoxF proteins (41, 44). Our data demonstrate that this regulation is different in *P. putida*, as *pedE* is only partially repressed by PedH. The fact that *pedE* repression in the presence of lanthanum is still observed in the Δ*pedH* mutant strain, together with the notion that the induction of *pedH* is not fully mediated by PedH, strongly suggests the existence of at least one additional lanthanum-responsive regulatory module. Notably, a very recent study found that the transmembrane-associated sensory histidine kinase MxaY mediates the lanthanide-responsive switch of the PQQ-dependent MDHs in *Methylomicrobium buryatense* (43). In *P. putida* KT2440, the genes for three different membrane-associated sensory histidine kinases, PedS1 (PP_2664), PedS2 (PP_2671), and PP_2683, are located in close proximity to *pedE* and *pedH* as part of the predicted ErbR (AgmR) regulon (17). Whether one of these sensor kinases serves a function similar to that of MxaY needs to be determined in future studies.

From an ecological point of view, it is interesting that growth and inverse regulation occur even in the presence of high Ca^2+^ concentrations (100 µM) when only nanomoles of lanthanum are supplied. From these data we conclude, similar to previous studies with *M. buryatense* or *M. extorquens* (32, 41), that the lanthanide-dependent enzyme PedH is the preferred PQQ-ADH when both metal cofactors are simultaneously accessible. Nevertheless, it was also demonstrated that at certain low REM concentrations, specific conditions exist under which both single mutants can grow. This suggests that the inverse regulation of the two enzymes is not a strict on-off switch but rather operates by strongly shifting transcription in favor of one of the enzymes, depending on the REM concentration.

REM-utilizing PQQ-ADHs have been suggested to be ancestral and more widespread than their calcium-dependent homologs (15, 50). This might indicate that calcium-dependent enzymes have evolved to colonize different and/or additional environmental niches in which lanthanide availability is less pronounced. Compared to soil environments, especially the rhizosphere, lanthanide concentrations in the phyllosphere and endosphere, as well as in other nonplant higher organisms, are comparably low (51–54). It is thus tempting to speculate that Ca^2+^-dependent enzymes are of particular relevance for interactions with multicellular organisms outside soil environments.

Metabolic interdependencies have been proposed as driving forces for species co-occurrence and the emergence of mutualism in diverse microbial communities, impacting their robustness, structure, and function (55–57). This is of particular interest in the context of periplasmic PQQ-ADHs, as organic alcohols and the corresponding oxidation products not only are crucial intermediates of the global carbon cycle but can also exhibit additional functions, including signaling and growth inhibition (4–7, 58). A recent study reported that regulation of the MxaF- and XoxF-type MDHs in a methanotrophic bacterium can be influenced by the presence of a nonmethanotrophic methylotroph in coculture experiments (59). The authors nicely demonstrate that during cocultivation in the presence of methane and lanthanides, the methanotrophic bacterium shifts its gene expression from the *xoxF*-type to the *mxaF*-type MDH. As a result of this change, leakage of methanol from the methanotroph was observed, which subsequently served as a growth substrate for the nonmethanotrophic partner. Although the mechanism of this phenomenon is not yet resolved, it indicates that different types of PQQ-ADHs might not only be important for potential interactions with higher organisms, as discussed above, but also within microbial communities. On the basis of our data, one can speculate that similar interactions are not limited to methano- and methylotrophic bacteria but are relevant in a much broader ecological context.

The discovery of lanthanides as a cofactor in biotechnologically important organisms other than methylotrophic bacteria expands the possible applications one can envision for REM biomining, bioleaching, and recycling processes (60–65). We believe that future research on lanthanide-utilizing enzymes and organisms will improve our understanding of natural and synthetic microbial communities and could provide a basis for novel biotechnological tools and processes.

## MATERIALS AND METHODS

### Bacterial strains, plasmids, and culture conditions.

For the strains and plasmids used in this study and a detailed description of their construction, see Text S1. Unless otherwise noted, *P. putida* KT2440 and *E. coli* strains were maintained on solidified (1.5% [wt/vol] agar) LB medium (66). Strains were routinely cultured in liquid LB medium, a modified M9 salt medium containing 68 mM phosphate buffer (pH 7), 18.6 mM NH_4_Cl, 8.6 mM NaCl, 2 mM MgSO_4_, and 100 µM CaCl_2_ with a trace element solution containing Na_3_-citrate at 51 µM, ZnSO_4_ at 7 µM, MnCl_2_ at 5 µM, CuSO_4_ at 4 µM, FeSO_4_ at 36 µM, H_3_BO_3_ at 5 µM, NaMoO_4_ at 137 nM, and NiCl_2_ at 84 nM or modified MP medium (67) containing 100 instead of 20 µM CaCl_2_ supplemented with succinate or 2-phenylethanol as the sole source of carbon and energy at 30°C with shaking. Where indicated, 40 µg ml^−1^ kanamycin or 15 µg ml^−1^ gentamicin for *E. coli* or 40 µg ml^−1^ kanamycin, 20 µg ml^−1^ 5-fluorouracil, or 30 µg ml^−1^ gentamicin for *P. putida* strains was added to the medium for maintenance and selection, respectively.

10.1128/mBio.00570-17.1 TEXT S1  Supplemental materials and methods, including the strains, plasmids, and primer used in this study. Download TEXT S1, DOCX file, 0.04 MB. Copyright © 2017 Wehrmann et al. 2017 Wehrmann et al. This content is distributed under the terms of the Creative Commons Attribution 4.0 International license.

### Liquid medium growth experiments.

All liquid medium growth experiments were carried out with modified M9 minimal salt medium or MP medium (see above) supplemented with 25 mM succinate or 5 mM 2-phenylethanol as a carbon and energy source. To avoid potential lanthanide contamination from glassware, all growth experiments were carried out with 125-ml polycarbonate vessels (Corning) or 96-well polypropylene plates with 2-ml-deep wells (Carl Roth). If not stated otherwise, precultures were grown in 5 ml of minimal medium (15-ml Falcon tubes) supplemented with succinate at 30°C and 180 rpm on a rotary shaker (Minitron; Infors HT). The next day, cultures were washed three times in fresh minimal medium without a carbon and energy source and used to inoculate 1 ml (for plates with 2-ml-deep wells) or 25 ml (for 125-ml polycarbonate vessels) of fresh medium to an initial OD at 600 nm (OD_600_) of 0.01. Subsequently, cultures were supplemented with the carbon and energy source, as well as various concentrations of lanthanides, and incubated at 30°C and 180 rpm (for 125-ml polycarbonate vessels) or 800 rpm (for plates with 2-ml-deep wells). For experiments with 125-ml polycarbonate vessels, growth was monitored by measuring the OD_600_ at regular intervals with a photometer (BioPhotometer; Eppendorf). For experiments carried out with 2-ml-deep well plates, OD_600_ was determined after 24 or 48 h by measuring 200 µl of cell culture transferred to a microtiter plate (Greiner Bio-One) in a microplate reader (POLARstar Omega; BMG Labtech). All data are presented as the mean value of biological triplicates with error bars representing the corresponding standard deviation.

### Agar plate assay.

For growth on solidified medium (1.5% [wt/vol] agar) with different substrates, M9 medium plates without a carbon source or trace element solution added were freshly prepared with or without the addition of 20 µM lanthanum chloride. Cell mass of the strains was obtained from LB agar plates, suspended in M9 medium without a carbon and energy source, and adjusted to an OD of 0.5. After the plates were dried for 20 min in a laminar-flow cabinet, 10 µl of each cell suspension was dropped onto the same plate and distributed with an inoculation loop over about 1/6 of each plate’s surface. When all strains were distributed, a 10-µl drop of a 1:1 (vol/vol) mixture of ethanol, 1-butanol, 2,3-butanediol, 1-octanol, or 2-phenylethanol in dimethyl sulfoxide (DMSO) was placed in the middle of the plate. Subsequently, the plates were sealed in plastic bags and incubated at room temperature. After 48 h, growth was quantified with a digital imaging system (Vilber Lourmat QUANTUM ST4) at the standard fluorescence settings with combined white light and UV illumination (excitation wavelength, 254 nm) for 1 s, an aperture of 11, and the preinstalled F590-nm filter. All individual pictures were subsequently sized, isolated from the background, and corrected for sharpness (+50%), brightness (+20%), and contrast (+40%) with the graphic formatting function in Microsoft PowerPoint.

### Transcriptional reporter assays.

For transcriptional reporter assays, *P. putida* harboring a Tn*7*-based *pedE-lux* or *pedH-lux* transcriptional reporter fusion was grown overnight in modified M9 or MP medium with succinate, washed three times in MP medium with no added carbon source, and finally suspended to an OD_600_ of 0.1 in MP medium or M9 medium with 1 mM 2-phenylethanol. For luminescence measurements, 180 μl of cell suspension was added to 20 μl of a 10-fold-concentrated metal salt solution in white 96-well plates with a clear bottom (µClear; Greiner Bio-One). Microtiter plates were placed in a humid box to prevent evaporation and incubated at 30°C with continuous agitation (200 rpm), and light emission and OD_600_ were recorded at regular intervals in an FLX-Xenius plate reader (SAFAS, Monaco) for up to 6 h. For both parameters, the background provided by the MP medium was subtracted and the luminescence was normalized to the corresponding OD_600_. Experiments were performed with biological triplicates, and data are presented as the mean value with error bars representing the corresponding standard deviation.

### Enzymatic activity assays.

For details of the PedE and PedH production and purification procedure, see Text S1. Purified PedE and PedH enzyme activities were measured in 96-well microtiter plates (Greiner Bio-One) with a dye-linked colorimetric assay based on previous studies (24, 68). Under optimized conditions (Fig. S4 to S8), one well contained a total volume of 250 µl of assay solution supplemented with 100 mM Tris HCl (pH 8), 500 µM phenazine methosulfate (PMS), 150 µM 2,6-dichlorophenol indophenol (DCPIP), 25 mM imidazole, 1 mM CaCl_2_ for PedE or 1 µM PrCl_3_ for PedH, 1 µM PQQ for PedE or 50 µM PQQ for PedH, 12.5 µl of substrate, and 2.5 to 20 µg/ml enzyme. The reaction was started by addition of the substrate to the reaction mixture, and activity was calculated on the basis of the change in OD_600_ within the first minute upon substrate addition. The molar extinction coefficient of DCPIP was experimentally determined to be 24.1 cm^−1^ M^−1^ at pH 8 (Fig. S4). Because of substrate-independent background activity, the assay solution was incubated for 45 min without a substrate at 30°C prior to enzyme activity measurements. As activities were between 8- and 12-fold higher with imidazole than with ammonium chloride or ethylamine, imidazole was used in all experiments (Fig. S5). Negative-control reactions, including the potential effect of bovine serum albumin or an assay mixture without the addition of an enzyme, did not show any reduction of DCPIP under the conditions used (data not shown). All assays were performed in three replicates, and data are presented as the mean value with error bars representing the corresponding standard deviation.

10.1128/mBio.00570-17.5 FIG S4  The extinction coefficient (ε) of DCPIP was calculated as 24.1 cm^−1^ mM^−1^ from the linear relationship between the OD_600_ and the DCPIP concentration. Download FIG S4, TIF file, 0.8 MB. Copyright © 2017 Wehrmann et al. 2017 Wehrmann et al. This content is distributed under the terms of the Creative Commons Attribution 4.0 International license.

10.1128/mBio.00570-17.6 FIG S5  Relative activity of PedE with 10 mM ethanol as the substrate in the presence of 25 mM imidazole (1), 45 mM ammonium chloride (2), or 5 mM ethylamine (3). Activity is 8 and 12 times higher with 25 mM imidazole than with 45 mM ammonium chloride and 5 mM ethylamine, respectively. Download FIG S5, TIF file, 0.8 MB. Copyright © 2017 Wehrmann et al. 2017 Wehrmann et al. This content is distributed under the terms of the Creative Commons Attribution 4.0 International license.

10.1128/mBio.00570-17.7 FIG S6  Relative activities of PedE (A) and PedH (B) with 10 mM ethanol as the substrate in the presence of increasing concentrations of imidazole. For better visualization, data were fitted (dashed line for PedE and continuous line for PedH) to a one-site binding model by nonlinear regression. Download FIG S6, TIF file, 1.7 MB. Copyright © 2017 Wehrmann et al. 2017 Wehrmann et al. This content is distributed under the terms of the Creative Commons Attribution 4.0 International license.

10.1128/mBio.00570-17.8 FIG S7  Relative activity of PedH with 10 mM ethanol as the substrate in the presence of various concentrations of PMS. A PMS concentration of 500 mM was determined to be sufficient for robust results by visual inspection. Download FIG S7, TIF file, 2.5 MB. Copyright © 2017 Wehrmann et al. 2017 Wehrmann et al. This content is distributed under the terms of the Creative Commons Attribution 4.0 International license.

10.1128/mBio.00570-17.9 FIG S8  Relative activities of PedE (A) and PedH (B) with 10 mM ethanol as the substrate in the presence of increasing concentrations of PQQ. Dashed (PedE) and continuous (PedH) lines represent a nonlinear fit to a one-site binding model. The *K*_*d*_ of 3.5 µM for PedE and 41 nM for PedH were calculated. Download FIG S8, TIF file, 1.7 MB. Copyright © 2017 Wehrmann et al. 2017 Wehrmann et al. This content is distributed under the terms of the Creative Commons Attribution 4.0 International license.

10.1128/mBio.00570-17.10 FIG S9  Growth of KT2440* (black circles), a Δ*pedE* strain (black diamonds), a Δ*pedH* strain (black squares), and a Δ*pedE* Δ*pedH* strain (black triangles) in liquid MP medium with 5 mM 2-phenylethanol in the presence of different La^3+^ concentrations. Growth was determined as the OD_600_ after incubation at 30°C for 48 h. Data are presented as the mean values of biological triplicates, and error bars represent the corresponding standard deviations. Download FIG S9, TIF file, 0.9 MB. Copyright © 2017 Wehrmann et al. 2017 Wehrmann et al. This content is distributed under the terms of the Creative Commons Attribution 4.0 International license.

### Metal dependency of the enzymes.

To test the metal dependency of PedE and PedH, a setup similar to that described above was used without CaCl_2_ for PedE or PrCl_3_ for PedH in the assay solution. Different REMs at 1 mM were added prior to incubation at 30°C. These included LaCl_3_, CeCl_3_, PrCl_3_, NdCl_3_, SmCl_3_, GdCl_3_, ErCl_3_, YbCl_3_, ScCl_3_, and YCl_3_. Activities were determined in triplicate as described above.

### Enzyme kinetics.

The kinetic constants of the enzyme substrate combinations were determined with the enzyme assay described above with various substrate concentrations measured in triplicate. The resulting activity constants were calculated by fitting the enzyme activities by nonlinear regression to the Michaelis-Menten equation by the Michaelis-Menten least-square fit method with no constrains in GraphPad Prism version 7.03 (GraphPad Software, Inc.).

### Homology models.

The PedE and PedH homology models were built with Swiss-Model (69). As ExaA has the highest sequence similarity to both PedE (60%) and PedH (49%) of all available crystal structures in the Swiss-Model template library, the crystal structure of ExaA, the PQQ-ADH of *P. aeruginosa* (PDB code 1FLG), was used as a template for model construction (23). Visualization of the models was carried out with PyMOL (70).

## References

[1] Insam H, Seewald MSA 2010 Volatile organic compounds (VOCs) in soils. Biol Fertil Soils 46:199–213. doi:10.1007/s00374-010-0442-3.

[2] Peñuelas J, Asensio D, Tholl D, Wenke K, Rosenkranz M, Piechulla B, Schnitzler JP 2014 Biogenic volatile emissions from the soil. Plant Cell Environ 37:1866–1891. doi:10.1111/pce.12340. 24689847

[3] van Dam NM, Weinhold A, Garbeva P 2016 Calling in the dark: the role of volatiles for communication in the rhizosphere, p 175–210. *In* Blande JD, Glinwood R (ed), Deciphering chemical language of plant communication. Springer International, Cham, Switzerland. doi:10.1007/978-3-319-33498-1_8.

[4] Schmidt R, Cordovez V, de Boer W, Raaijmakers J, Garbeva P 2015 Volatile affairs in microbial interactions. ISME J 9:2329–2335. doi:10.1038/ismej.2015.42. 26023873 PMC4611499

[5] Jones SE, Elliot MA 2017 Streptomyces exploration: competition, volatile communication and new bacterial behaviours. Trends Microbiol 26:3167–3170. doi:10.1016/j.tim.2017.02.001. 28245952

[6] Tyc O, Song C, Dickschat JS, Vos M, Garbeva P 2017 The ecological role of volatile and soluble secondary metabolites produced by soil bacteria. Trends Microbiol 25:280–292. doi:10.1016/j.tim.2016.12.002. 28038926

[7] Bitas V, Kim HS, Bennett JW, Kang S 2013 Sniffing on microbes: diverse roles of microbial volatile organic compounds in plant health. Mol Plant Microbe Interact 26:835–843. doi:10.1094/MPMI-10-12-0249-CR. 23581824

[8] Adachi O, Ano Y, Toyama H, Matsushita K 2007 Biooxidation with PQQ- and FAD-dependent dehydrogenases, p 1–41. *In* Schmid RD, Urlacher VB (ed), Modern biooxidation: enzymes, reactions and applications. Wiley-VCH Verlag GmbH & Co. KGaA, Weinheim, Germany. doi:10.1002/9783527611522.ch1.

[9] Toyama H, Mathews FS, Adachi O, Matsushita K 2004 Quinohemoprotein alcohol dehydrogenases: structure, function, and physiology. Arch Biochem Biophys 428:10–21. doi:10.1016/j.abb.2004.03.037. 15234265

[10] Anthony C, Ghosh M, Blake CCF 1994 The structure and function of methanol dehydrogenase and related quinoproteins containing pyrrolo-quinoline quinone. Biochem J 304:665–674. doi:10.1042/bj3040665. 7818466 PMC1137385

[11] Oubrie A, Dijkstra BW 2000 Structural requirements of pyrroloquinoline quinone dependent enzymatic reactions. Protein Sci 9:1265–1273. doi:10.1110/ps.9.7.1265. 10933491 PMC2144678

[12] Kay CWM, Mennenga B, Görisch H, Bittl R 2005 Substrate-binding in quinoprotein ethanol dehydrogenase from *Pseudomonas aeruginosa* studied by electron paramagnetic resonance at 94 GHz. J Am Chem Soc 127:7974–7975. doi:10.1021/ja050972c. 15926796

[13] Anthony C 2001 Pyrroloquinoline quinone (PQQ) and quinoprotein enzymes. Antioxid Redox Signal 3:757–774. doi:10.1089/15230860152664966. 11761326

[14] Toyama H, Fujii A, Matsushita K, Shinagawa E, Ameyama M, Adachi O 1995 Three distinct quinoprotein alcohol dehydrogenases are expressed when *Pseudomonas putida* is grown on different alcohols. J Bacteriol 177:2442–2450. doi:10.1128/jb.177.9.2442-2450.1995. 7730276 PMC176903

[15] Keltjens JT, Pol A, Reimann J, Op den Camp HJM 2014 PQQ-dependent methanol dehydrogenases: rare-earth elements make a difference. Appl Microbiol Biotechnol 98:6163–6183. doi:10.1007/s00253-014-5766-8. 24816778

[16] Good NM, Vu HN, Suriano CJ, Subuyuj GA, Skovran E, Martinez-Gomez NC 2016 Pyrroloquinoline quinone ethanol dehydrogenase in *Methylobacterium extorquens* AM1 extends lanthanide-dependent metabolism to multicarbon substrates. J Bacteriol 198:3109–3118. doi:10.1128/JB.00478-16. 27573017 PMC5075040

[17] Mückschel B, Simon O, Klebensberger J, Graf N, Rosche B, Altenbuchner J, Pfannstiel J, Huber A, Hauer B 2012 Ethylene glycol metabolism by *Pseudomonas putida*. Appl Environ Microbiol 78:8531–8539. doi:10.1128/AEM.02062-12. 23023748 PMC3502918

[18] Takeda K, Matsumura H, Ishida T, Samejima M, Igarashi K, Nakamura N, Ohno H 2013 The two-step electrochemical oxidation of alcohols using a novel recombinant PQQ alcohol dehydrogenase as a catalyst for a bioanode. Bioelectrochemistry 94:75–78. doi:10.1016/j.bioelechem.2013.08.001. 24036413

[19] Simon O, Klebensberger J, Mükschel B, Klaiber I, Graf N, Altenbuchner J, Huber A, Hauer B, Pfannstiel J 2015 Analysis of the molecular response of *Pseudomonas putida* KT2440 to the next-generation biofuel n-butanol. J Proteomics 122:11–25. doi:10.1016/j.jprot.2015.03.022. 25829261

[20] Kay CWM, Mennenga B, Görisch H, Bittl R 2006 Substrate binding in quinoprotein ethanol dehydrogenase from *Pseudomonas aeruginosa* studied by electron-nuclear double resonance. Proc Natl Acad Sci U S A 103:5267–5272. doi:10.1073/pnas.0509667103. 16567634 PMC1459345

[21] Mennenga B, Kay CWM, Görisch H 2009 Quinoprotein ethanol dehydrogenase from *Pseudomonas aeruginosa*: the unusual disulfide ring formed by adjacent cysteine residues is essential for efficient electron transfer to cytochrome c550. Arch Microbiol 191:361–367. doi:10.1007/s00203-009-0460-4. 19224199

[22] Kay CWM, Mennenga B, Görisch H, Bittl R 2006 Structure of the pyrroloquinoline quinone radical in quinoprotein ethanol dehydrogenase. J Biol Chem 281:1470–1476. doi:10.1074/jbc.M511132200. 16267040

[23] Keitel T, Diehl A, Knaute T, Stezowski JJ, Höhne W, Görisch H 2000 X-ray structure of the quinoprotein ethanol dehydrogenase from *Pseudomonas aeruginosa*: basis of substrate specificity. J Mol Biol 297:961–974. doi:10.1006/jmbi.2000.3603. 10736230

[24] Chattopadhyay A, Förster-Fromme K, Jendrossek D 2010 PQQ-dependent alcohol dehydrogenase (QEDH) of *Pseudomonas aeruginosa* is involved in catabolism of acyclic terpenes. J Basic Microbiol 50:119–124. doi:10.1002/jobm.200900178. 20082374

[25] Williams PA, Coates L, Mohammed F, Gill R, Erskine PT, Coker A, Wood SP, Anthony C, Cooper JB 2005 The atomic resolution structure of methanol dehydrogenase from *Methylobacterium extorquens*. Acta Crystallogr D Biol Crystallogr 61:75–79. doi:10.1107/S0907444904026964. 15608378

[26] Chistoserdova L, Lidstrom ME 1997 Molecular and mutational analysis of a DNA region separating two methylotrophy gene clusters in *Methylobacterium extorquens* AM1. Microbiology 143:1729–1736. doi:10.1099/00221287-143-5-1729. 9168622

[27] Richardson IW, Anthony C 1992 Characterization of mutant forms of the quinoprotein methanol dehydrogenase lacking an essential calcium ion. Biochem J 287:709–715. doi:10.1042/bj2870709. 1332681 PMC1133066

[28] Nakagawa T, Mitsui R, Tani A, Sasa K, Tashiro S, Iwama T, Hayakawa T, Kawai K 2012 A catalytic role of XoxF1 as La^3+^-dependent methanol dehydrogenase in *Methylobacterium extorquens* strain AM1. PLoS One 7:e50480. doi:10.1371/journal.pone.0050480. 23209751 PMC3507691

[29] Hibi Y, Asai K, Arafuka H, Hamajima M, Iwama T, Kawai K 2011 Molecular structure of La^3+^-induced methanol dehydrogenase-like protein in *Methylobacterium radiotolerans*. J Biosci Bioeng 111:547–549. doi:10.1016/j.jbiosc.2010.12.017. 21256798

[30] Fitriyanto NA, Fushimi M, Matsunaga M, Pertiwiningrum A, Iwama T, Kawai K 2011 Molecular structure and gene analysis of Ce^3+^-induced methanol dehydrogenase of *Bradyrhizobium sp.* MAFF211645. J Biosci Bioeng 111:613–617. doi:10.1016/j.jbiosc.2011.01.015. 21334970

[31] Wu ML, Wessels JCT, Pol A, Op den Camp HJM, Jetten MSM, van Niftrik L 2015 XoxF-type methanol dehydrogenase from the anaerobic methanotroph “*Candidatus* Methylomirabilis oxyfera”. Appl Environ Microbiol 81:1442–1451. doi:10.1128/AEM.03292-14. 25527536 PMC4309699

[32] Chu F, Lidstrom ME 2016 XoxF acts as the predominant methanol dehydrogenase in the type I methanotroph *Methylomicrobium buryatense*. J Bacteriol 198:1317–1325. doi:10.1128/JB.00959-15. 26858104 PMC4859581

[33] Pol A, Barends TRM, Dietl A, Khadem AF, Eygensteyn J, Jetten MSM, Op den Camp HJM 2014 Rare earth metals are essential for methanotrophic life in volcanic mudpots. Environ Microbiol 16:255–264. doi:10.1111/1462-2920.12249. 24034209

[34] Taubert M, Grob C, Howat AM, Burns OJ, Dixon JL, Chen Y, Murrell JC 2015 XoxF encoding an alternative methanol dehydrogenase is widespread in coastal marine environments. Environ Microbiol 17:3937–3948. doi:10.1111/1462-2920.12896. 25943904

[35] Skovran E, Martinez-Gomez NC 2015 Microbiology. Just add lanthanides. Science 348:862–863. doi:10.1126/science.aaa9091. 25999492

[36] Martinez-Gomez NC, Vu HN, Skovran E 2016 Lanthanide chemistry: from coordination in chemical complexes shaping our technology to coordination in enzymes shaping bacterial metabolism. Inorg Chem 55:10083–10089. doi:10.1021/acs.inorgchem.6b00919. 27588435

[37] Nikel PI, Martínez-García E, de Lorenzo V 2014 Biotechnological domestication of pseudomonads using synthetic biology. Nat Rev Microbiol 12:368–379. doi:10.1038/nrmicro3253. 24736795

[38] Loeschcke A, Thies S 2015 *Pseudomonas putida*—a versatile host for the production of natural products. Appl Microbiol Biotechnol 99:6197–6214. doi:10.1007/s00253-015-6745-4. 26099332 PMC4495716

[39] Nikel PI, Chavarría M, Danchin A, de Lorenzo V 2016 From dirt to industrial applications: *Pseudomonas putida* as a synthetic biology chassis for hosting harsh biochemical reactions. Curr Opin Chem Biol 34:20–29. doi:10.1016/j.cbpa.2016.05.011. 27239751

[40] Poblete-Castro I, Becker J, Dohnt K, dos Santos VM, Wittmann C 2012 Industrial biotechnology of *Pseudomonas putida* and related species. Appl Microbiol Biotechnol 93:2279–2290. doi:10.1007/s00253-012-3928-0. 22350258

[41] Vu HN, Subuyuj GA, Vijayakumar S, Good NM, Martinez-Gomez NC, Skovran E 2016 Lanthanide-dependent regulation of methanol oxidation systems in *Methylobacterium extorquens* AM1 and their contribution to methanol growth. J Bacteriol 198:1250–1259. doi:10.1128/JB.00937-15. 26833413 PMC4859578

[42] Farhan Ul Haque M, Kalidass B, Bandow N, Turpin EA, DiSpirito AA, Semrau JD 2015 Cerium regulates expression of alternative methanol dehydrogenases in *Methylosinus trichosporium* OB3b. Appl Environ Microbiol 81:7546–7552. doi:10.1128/AEM.02542-15. 26296730 PMC4592857

[43] Chu F, Beck DAC, Lidstrom ME 2016 MxaY regulates the lanthanide-mediated methanol dehydrogenase switch in *Methylomicrobium buryatense*. PeerJ 4:e2435. doi:10.7717/peerj.2435. 27651996 PMC5018670

[44] Skovran E, Palmer AD, Rountree AM, Good NM, Lidstrom ME 2011 XoxF is required for expression of methanol dehydrogenase in *Methylobacterium extorquens* AM1. J Bacteriol 193:6032–6038. doi:10.1128/JB.05367-11. 21873495 PMC3194914

[45] Shannon RD 1976 Revised effective ionic radii and systematic studies of interatomic distances in halides and chalcogenides. Acta Crystallogr A 32:751–767. doi:10.1107/S0567739476001551.

[46] Bogart JA, Lewis AJ, Schelter EJ 2015 DFT study of the active site of the XoxF-type natural, cerium-dependent methanol dehydrogenase enzyme. Chemistry 21:1743–1748. doi:10.1002/chem.201405159. 25421364

[47] Goodwin MG, Anthony C 1996 Characterization of a novel methanol dehydrogenase containing a Ba^2+^ ion at the active site. Biochem J 318:673–679. doi:10.1042/bj3180673. 8809062 PMC1217672

[48] Wagner A 2005 Distributed robustness versus redundancy as causes of mutational robustness. Bioessays 27:176–188. doi:10.1002/bies.20170. 15666345

[49] Arias S, Olivera ER, Arcos M, Naharro G, Luengo JM 2008 Genetic analyses and molecular characterization of the pathways involved in the conversion of 2-phenylethylamine and 2-phenylethanol into phenylacetic acid in *Pseudomonas putida* U. Environ Microbiol 10:413–432. doi:10.1111/j.1462-2920.2007.01464.x. 18177365

[50] Vekeman B, Speth D, Wille J, Cremers G, De Vos P, Op den Camp HJM, Heylen K 2016 Genome characteristics of two novel type I methanotrophs enriched from North Sea sediments containing exclusively a lanthanide-dependent XoxF5-type methanol dehydrogenase. Microb Ecol 72:503–509. doi:10.1007/s00248-016-0808-7. 27457652

[51] Markert B 1987 The pattern of distribution of lanthanide elements in soils and plants. Phytochemistry 26:3167–3170. doi:10.1016/S0031-9422(00)82463-2.

[52] Carpenter D, Boutin C, Allison JE, Parsons JL, Ellis DM 2015 Uptake and effects of six rare earth elements (REEs) on selected native and crop species growing in contaminated soils. PLoS One 10:e0129936. doi:10.1371/journal.pone.0129936. 26076480 PMC4468158

[53] Aubert D, Stille P, Probst A, Gauthier-lafaye F, Pourcelot L, Del nero M 2002 Characterization and migration of atmospheric REE in soils and surface waters. Geochim Cosmochim Acta 66:3339–3350. doi:10.1016/S0016-7037(02)00913-4.

[54] Kabata-Pendias A, Mukherjee AB 2007 Trace elements from soil to human. Springer, Berlin, Germany. doi:10.1007/978-3-540-32714-1.

[55] Estrela S, Brown SP 2013 Metabolic and demographic feedbacks shape the emergent spatial structure and function of microbial communities. PLoS Comput Biol 9:e1003398. doi:10.1371/journal.pcbi.1003398. 24385891 PMC3873226

[56] Zelezniak A, Andrejev S, Ponomarova O, Mende DR, Bork P, Patil KR 2015 Metabolic dependencies drive species co-occurrence in diverse microbial communities. Proc Natl Acad Sci U S A 112:6449–6454. doi:10.1073/pnas.1421834112. 25941371 PMC4443341

[57] LaSarre B, McCully AL, Lennon JT, McKinlay JB 2017 Microbial mutualism dynamics governed by dose-dependent toxicity of cross-fed nutrients. ISME J 11:337–348. doi:10.1038/ismej.2016.141. 27898053 PMC5270580

[58] Schink B 1997 Energetics of syntrophic cooperation in methanogenic degradation. Microbiol Mol Biol Rev 61:262–280. 9184013 10.1128/mmbr.61.2.262-280.1997 PMC232610

[59] Krause SMB, Johnson T, Samadhi Karunaratne Y, Fu Y, Beck DAC, Chistoserdova L, Lidstrom ME 2017 Lanthanide-dependent cross-feeding of methane-derived carbon is linked by microbial community interactions. Proc Natl Acad Sci U S A 114:358–363. doi:10.1073/pnas.1619871114. 28028242 PMC5240692

[60] Das N, Das D 2013 Recovery of rare earth metals through biosorption: an overview. J Rare Earths 31:933–943. doi:10.1016/S1002-0721(13)60009-5.

[61] Gu W, Farhan Ul Haque M, DiSpirito AA, Semrau JD 2016 Uptake and effect of rare earth elements on gene expression in *Methylosinus trichosporium* OB3b. FEMS Microbiol Lett 363:fnw129. doi:10.1093/femsle/fnw129. 27190151

[62] Emmanuel ES, Ananthi T, Anandkumar B, Maruthamuthu S 2012 Accumulation of rare earth elements by siderophore-forming *Arthrobacter luteolus* isolated from rare earth environment of Chavara, India. J Biosci 37:25–31. doi:10.1007/s12038-011-9173-3. 22357200

[63] Challaraj Emmanuel ES, Vignesh V, Anandkumar B, Maruthamuthu S 2011 Bioaccumulation of cerium and neodymium by *Bacillus cereus* isolated from rare earth environments of Chavara and Manavalakurichi, India. Indian J Microbiol 51:488–495. doi:10.1007/s12088-011-0111-8. 23024412 PMC3209947

[64] Barmettler F, Castelberg C, Fabbri C, Brandl H 2016 Microbial mobilization of rare earth elements (REE) from mineral solids—a mini review. AIMS Microbiol 2:190–204. doi:10.3934/microbiol.2016.2.190.

[65] Ene CD, Ruta LL, Nicolau I, Popa CV, Iordache V, Neagoe AD, Farcasanu IC 2015 Interaction between lanthanide ions and *Saccharomyces cerevisiae* cells. J Biol Inorg Chem 20:1097–1107. doi:10.1007/s00775-015-1291-1. 26267167

[66] Maniatis T, Fritsch EF, Sambrook J 1982 Molecular cloning: a laboratory manual. Cold Spring Harbor Laboratory, Cold Spring Harbor, NY.

[67] Delaney NF, Kaczmarek ME, Ward LM, Swanson PK, Lee MC, Marx CJ 2013 Development of an optimized medium, strain and high-throughput culturing methods for *Methylobacterium extorquens*. PLoS One 8:e62957. doi:10.1371/journal.pone.0062957. 23646164 PMC3639900

[68] Anthony C, Zatman LJ 1964 The microbial oxidation of methanol. 2. The methanol-oxidizing enzyme of Pseudomonas sp. M 27. Biochem J 92:614–621. 4378696 10.1042/bj0920614 PMC1206111

[69] Biasini M, Bienert S, Waterhouse A, Arnold K, Studer G, Schmidt T, Kiefer F, Gallo Cassarino TG, Bertoni M, Bordoli L, Schwede T 2014 SWISS-MODEL: modelling protein tertiary and quaternary structure using evolutionary information. Nucleic Acids Res 42:W252–W258. doi:10.1093/nar/gku340. 24782522 PMC4086089

[70] Schrödinger LLC. 2015 The PyMOL molecular graphics system, version 1.8. Schrödinger LLC, Cambridge, MA.

