# Supplementary figures and images for: Functional Role of Lanthanides in Enzymatic Activity and Transcriptional Regulation of Pyrroloquinoline Quinone-Dependent Alcohol Dehydrogenases in Pseudomonas putida KT2440
Source: mBio. 2017 Jun 27;8(3):e00570-17. doi: 10.1128/mBio.00570-17 (PMC5487730; doi:10.1128/mBio.00570-17)

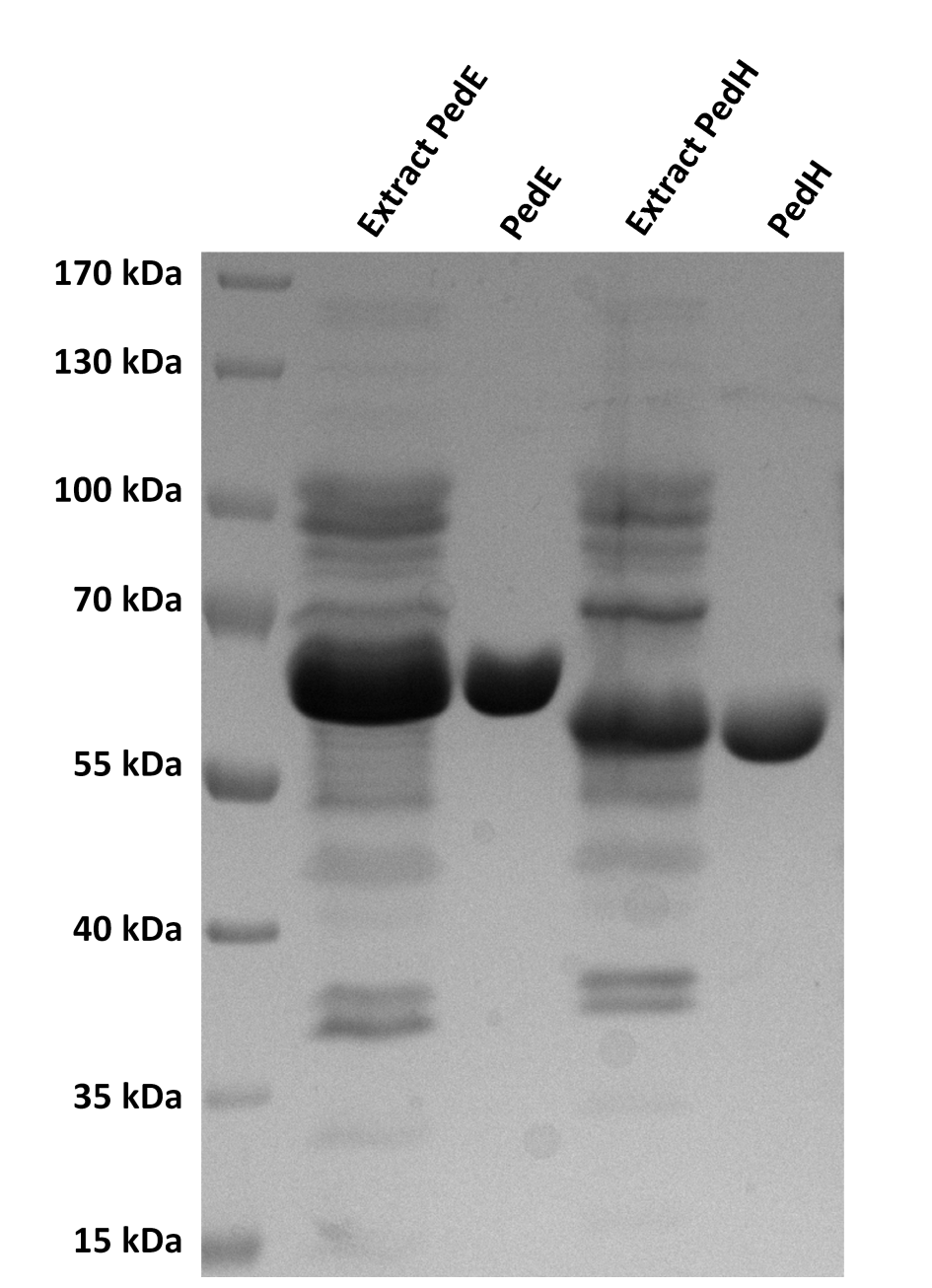

Supplement: FIG S1 [file mbo003173354sf1.tif]

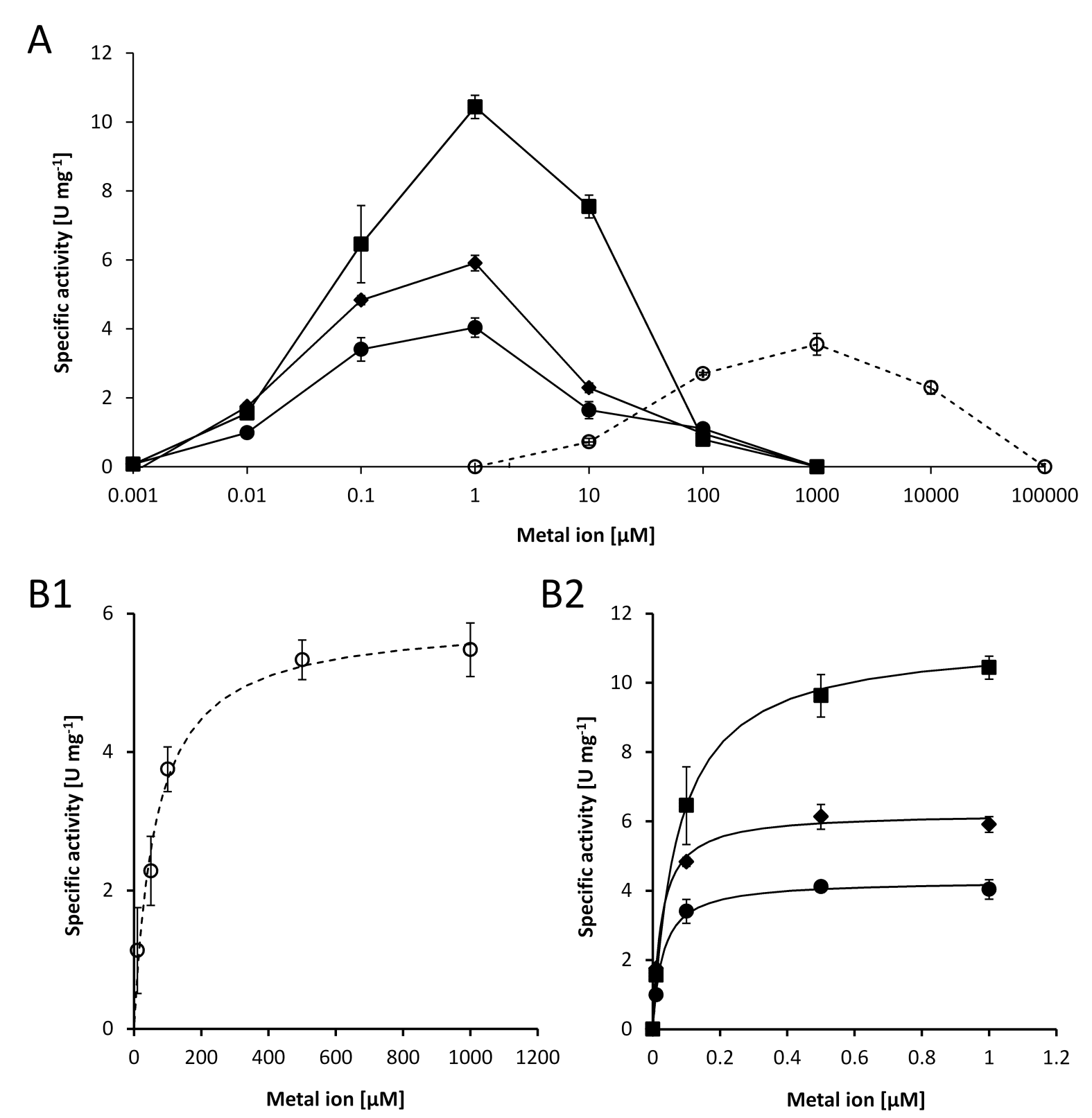

Supplement: FIG S2 [file mbo003173354sf2.tif]

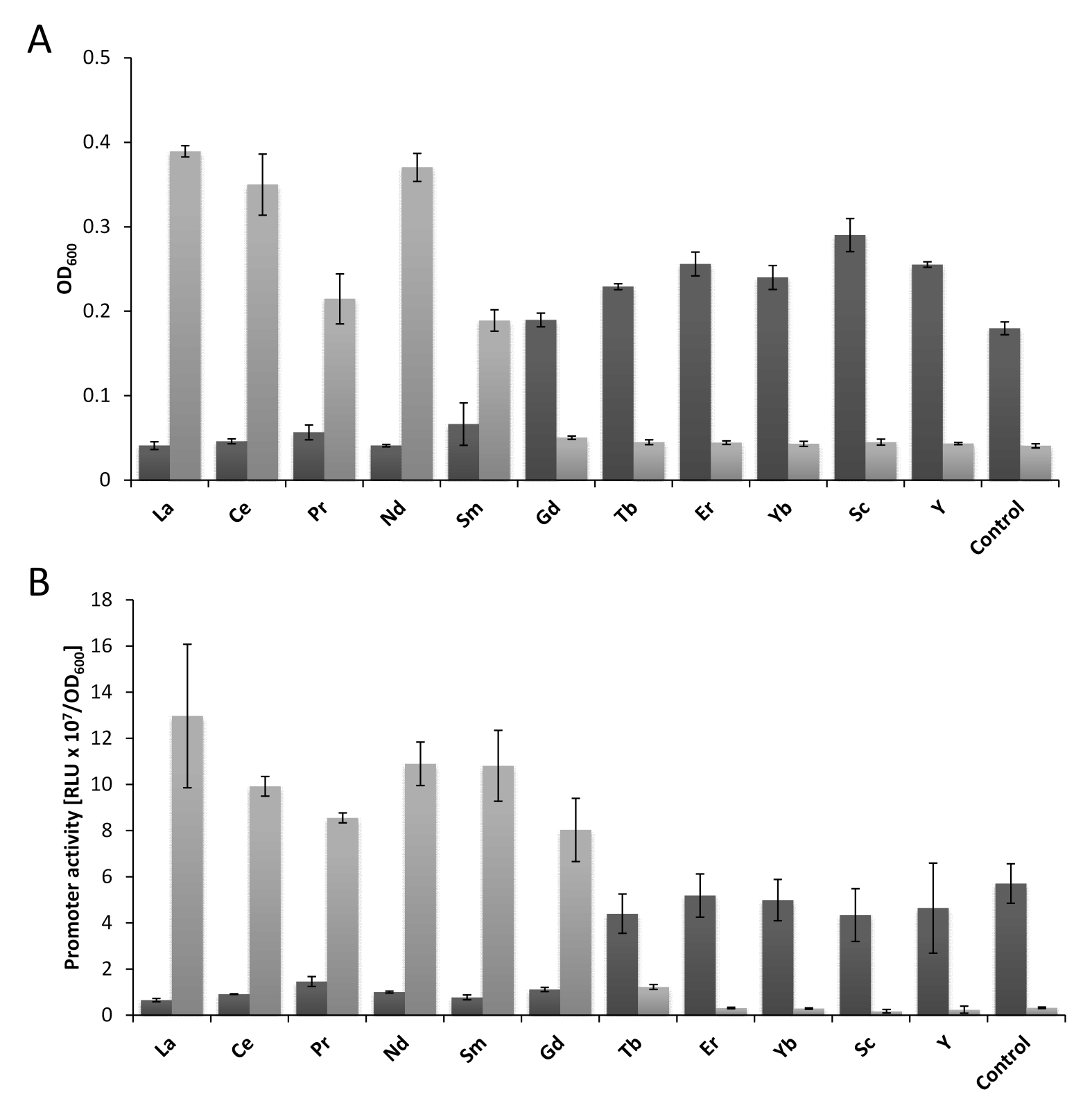

Supplement: FIG S3 [file mbo003173354sf3.tif]

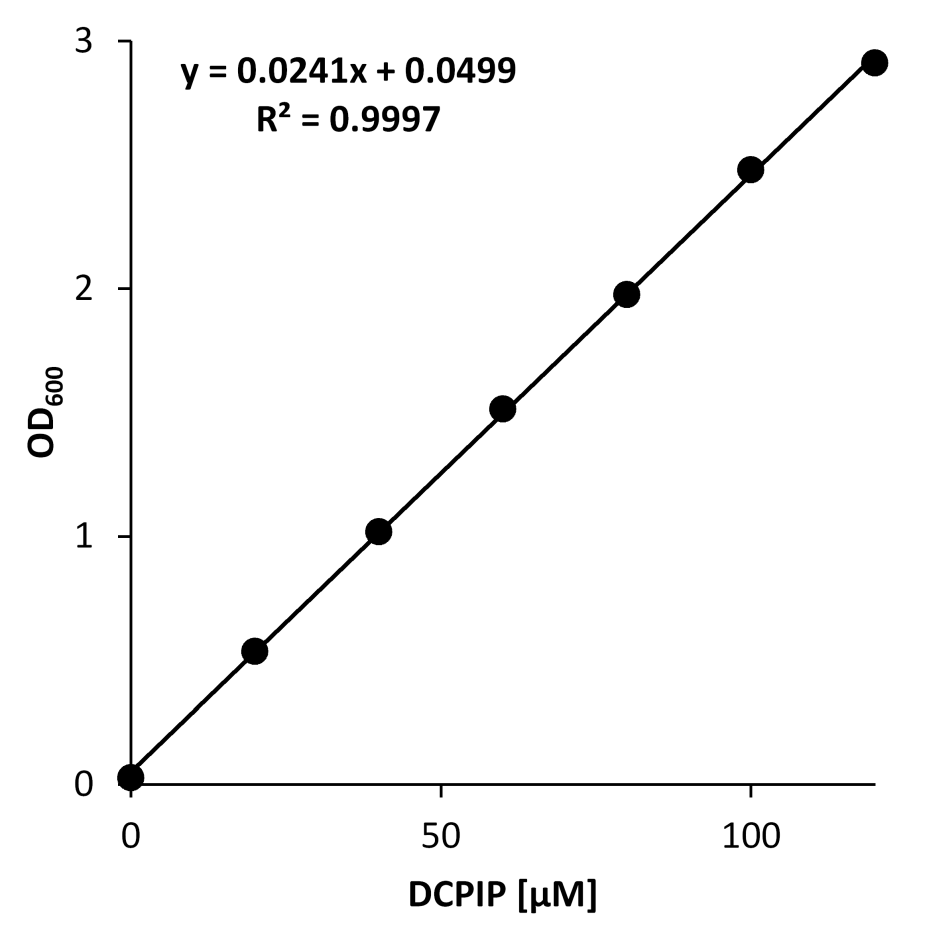

Supplement: FIG S4 [file mbo003173354sf4.tif]

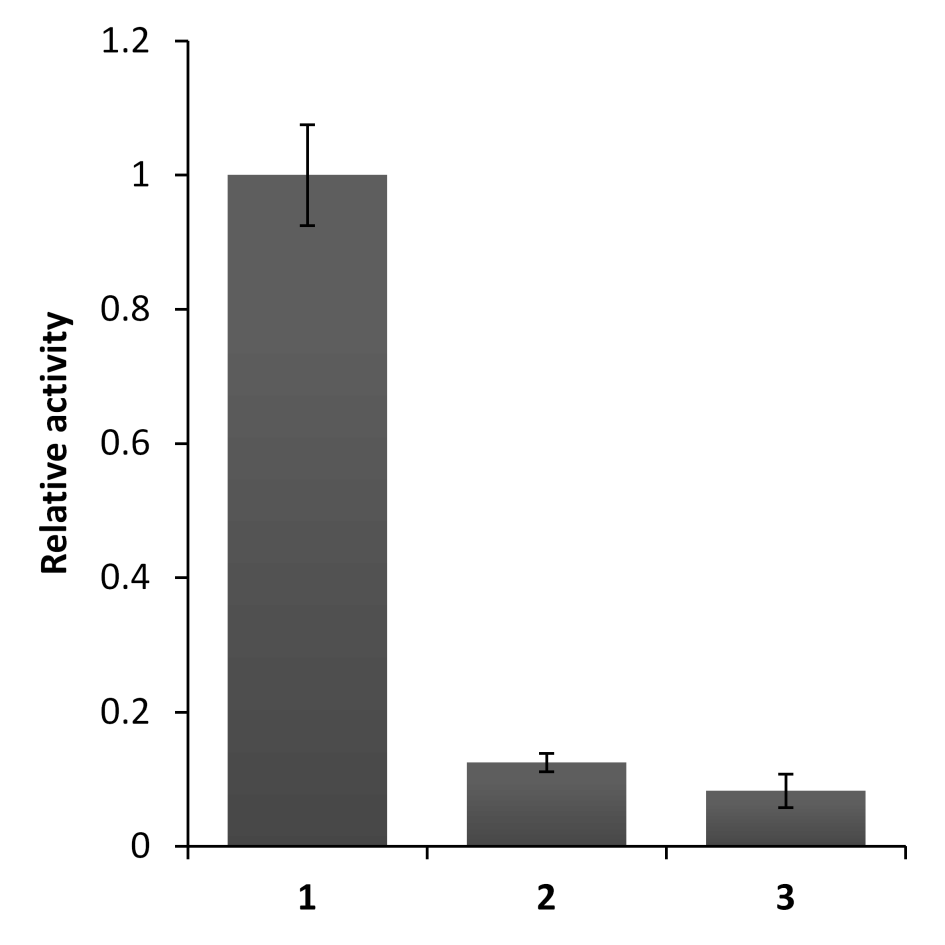

Supplement: FIG S5 [file mbo003173354sf5.tif]

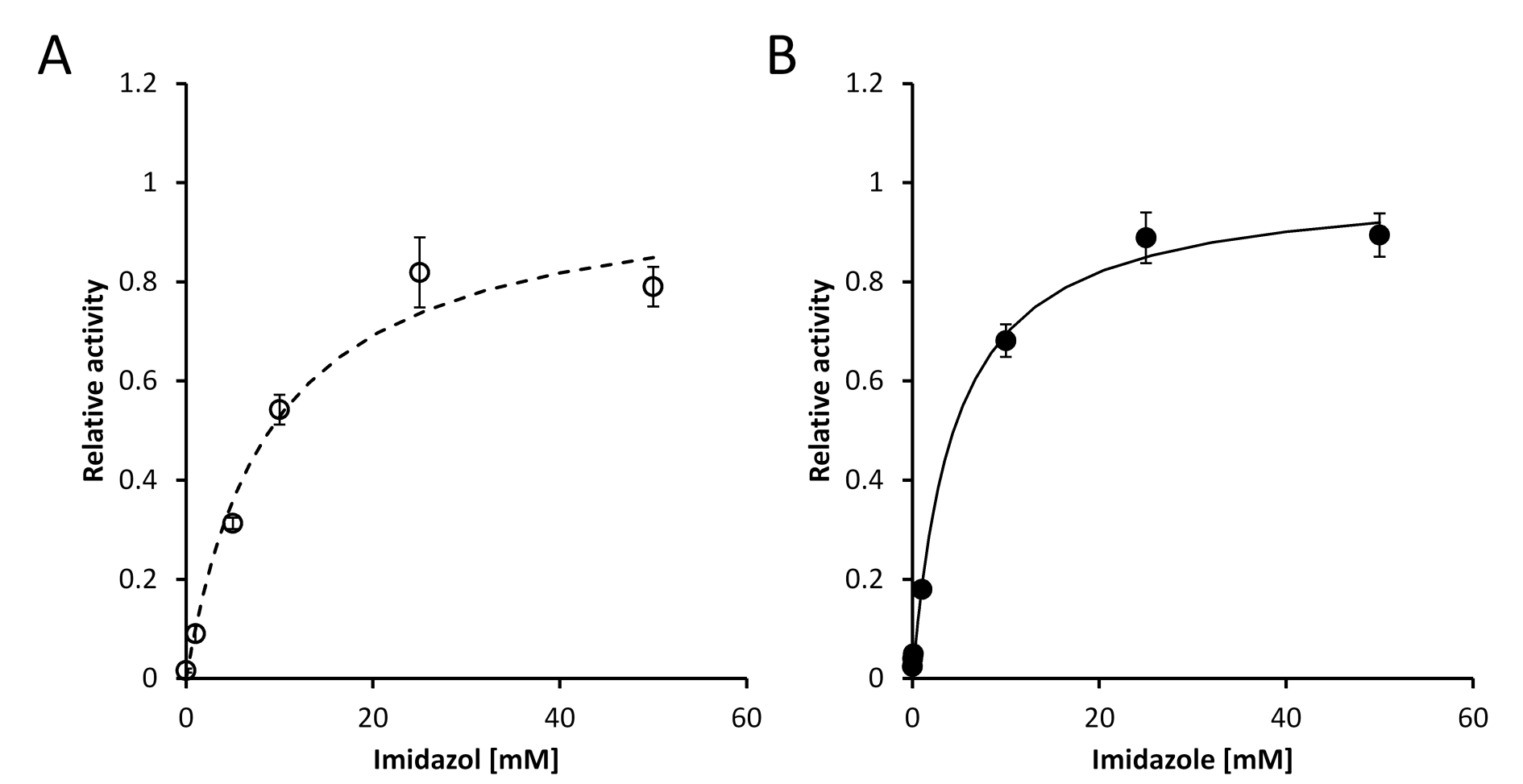

Supplement: FIG S6 [file mbo003173354sf6.tif]

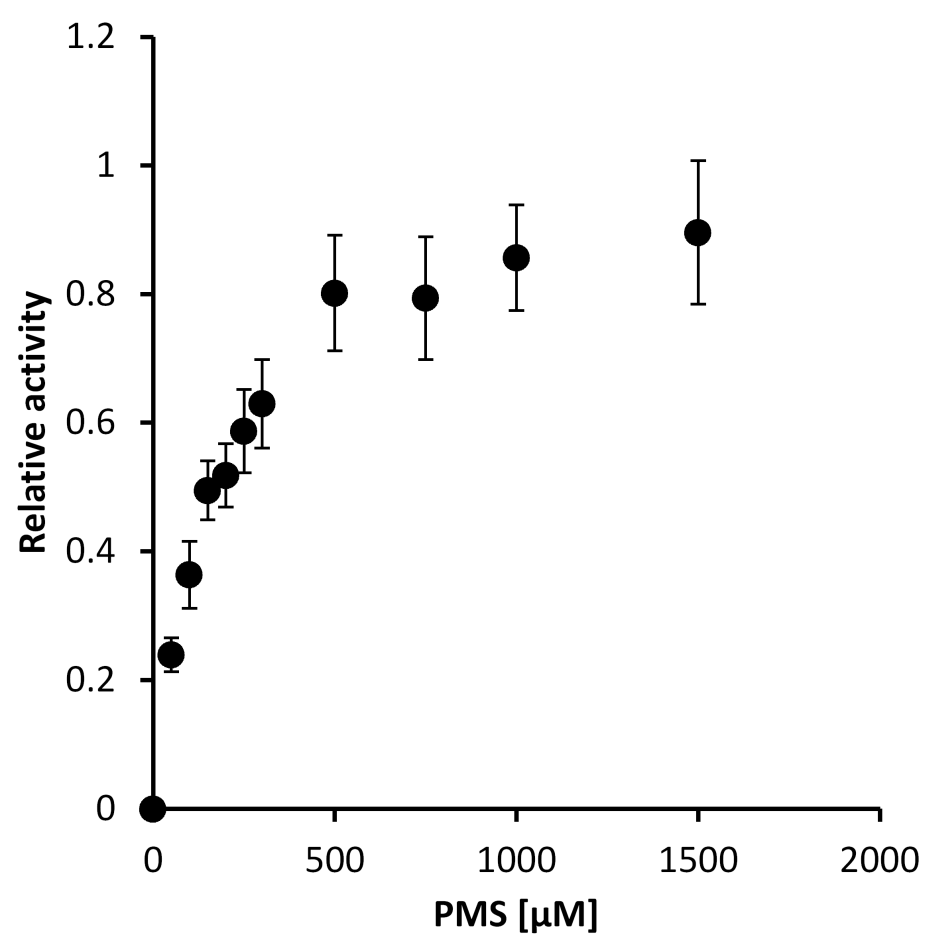

Supplement: FIG S7 [file mbo003173354sf7.tif]

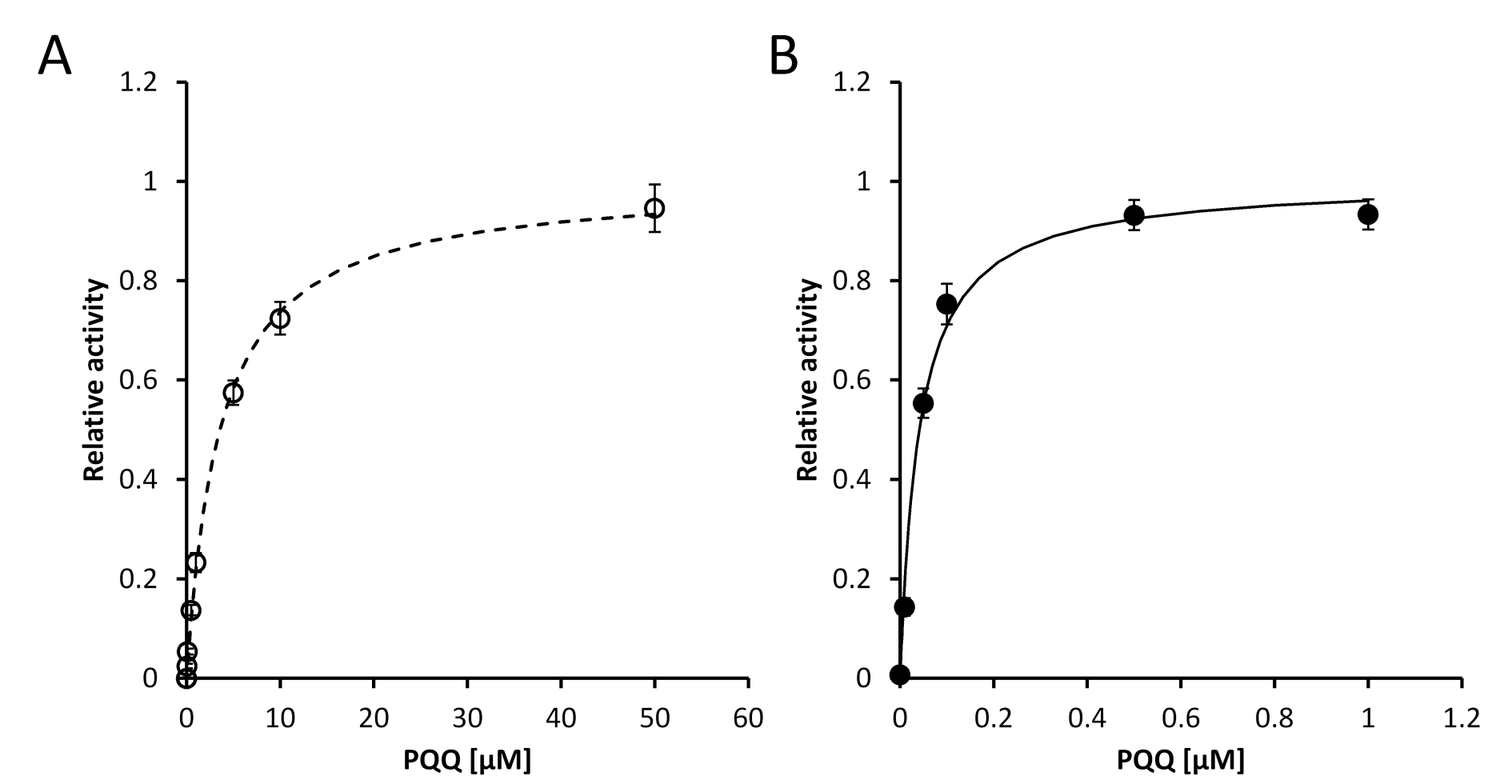

Supplement: FIG S8 [file mbo003173354sf8.tif]

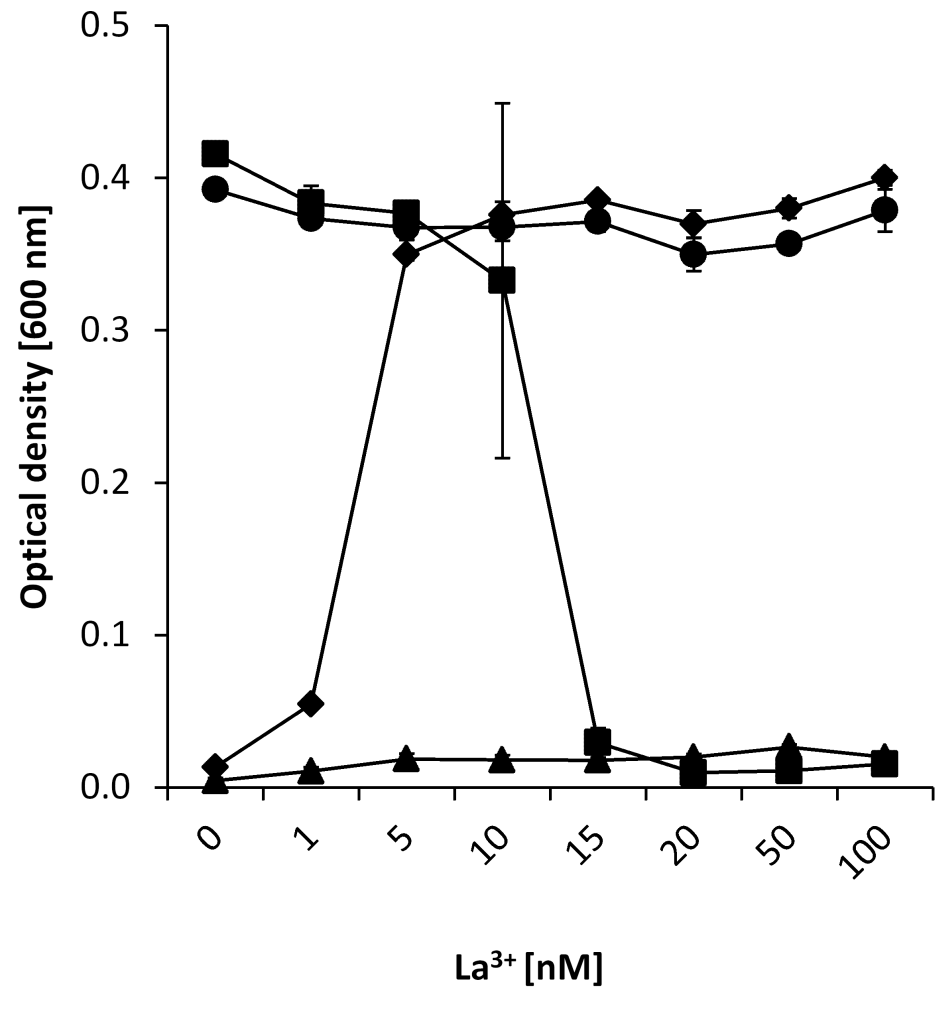

Supplement: FIG S9 [file mbo003173354sf9.tif]
