# Supplementary material for: Functional Role of Lanthanides in Enzymatic Activity and Transcriptional Regulation of Pyrroloquinoline Quinone-Dependent Alcohol Dehydrogenases in Pseudomonas putida KT2440
Source: mBio. 2017 Jun 27;8(3):e00570-17. doi: 10.1128/mBio.00570-17 (PMC5487730; doi:10.1128/mBio.00570-17)
Supplement: TEXT S1 [file mbo003173354s1.docx]

**Functional role of lanthanides in enzymatic activity and transcriptional regulation of PQQ-dependent alcohol dehydrogenases in *Pseudomonas putida* KT2440**

Matthias Wehrmann^a^, Patrick Billard^b,c^, Audrey Martin-Meriadec^b,c^, Asfaw Zegeye^b,c^, Janosch Klebensberger^a^#

***Specific chemicals***

Pyrroloquinoline quinone (PQQ) disodium salt, 2,6-dichlorophenol indophenol sodium salt and 5-fluorouracil were purchased from Sigma-Aldrich. Phenazine methosulfate was obtained from Santa Cruz Biotechnology. Lanthanum, praseodymium, cerium, neodymium, samarium, gadolinium, terbium, erbium, ytterbium, scandium and yttrium were obtained as chloride salts from Sigma-Aldrich or Santa Cruz Biotechnology. All liquid chemicals were purchased from VWR Chemicals, Merck or Sigma-Aldrich in purities of ≥ 99.0 %.

***Construction of plasmids***

The *pedE* and *pedH* genes of *Pseudomonas putida* KT2440 were amplified from genomic DNA using Q5 Hot-Start DNA polymerase (New England BioLabs) and primer pairs *MWH11*/*MWH12 and MWH13* /*MWH14* (**Table S1**). The primers harbored a C-terminal 6xHis-tag and a > 15 bp homology to the insertion sites in plasmid pJeM1 on each site. The *eGFP* gene in plasmid pJeM1 was excised using NdeI and HindIII and replaced with the purified PCR products *via* the one-step isothermal assembly as described by Gibson *et al.* (1)*.* The constructs were subsequently transformed into *E. coli* BL21 (DE3) and the correctness of the cloned genes was verified by Sanger sequencing (GATC Biotech).

For construction of the integration vector pMW43, pJOE6261.2 was digested with BamHI. Additionally the 1000 bp regions up- and downstream of the gene cluster *pqqABCDE* were amplified from genomic DNA of *P. putida* KT2440 using primer pairs MWH34/MWH35 and MWH36/MWH37. The three fragments were joined together using the one-step isothermal assembly as described by Gibson *et al.* (1). The constructs were subsequently transformed into *E. coli* BL21 (DE3) and the correctness of the plasmid was confirmed by Sanger sequencing.

For quantifying the transcriptional activities of *pedE* and *pedH* *in vivo* plasmids pUC18-mini-Tn*7*-*pedE*-*lux*-Gm and pUC18-mini-Tn*7*T-*pedH*-*lux*-Gm were constructed (2). The DNA regions encompassing the promoter from *pedE and pedH* genes were amplified by PCR (Phusion DNA polymerase, Fermentas) using the primer pairs p2674-FSac/p2674-RPst and p2679-FSac/p2679-RPst (**Table S1**), respectively*.* PCR products were digested with SacI and PstI and cloned upstream the *luxCDABE* operon hosted by plasmid pUC18-mini-Tn*7*T-Gm-lux. The resulting mini-Tn7 constructs were co-electroporated with the helper plasmid pTNS2 into KT2440 and mutant strains ∆*pedE* and ∆*pedH* (2, 3). Proper chromosomal integration of Tn7 elements was verified by PCR using Pput-*glmS*DN and PTn*7*R primers as described previously (2).

**Table 1:** Strain, plasmids, and primer used in the study

| **Strains** | **Relevant features** | |
| --- | --- | --- |
| KT2440 | Wildtype strain of *Pseudomonas putida* (ATCC 47054) | |
| KT2440* | KT2440 with a markerless deletion of *upp* \| Parent strain for deletion mutants (4) | |
| ∆*pedH* | ∆*upp* with a markerless mutation of *pedH* (5) | |
| ∆*pedE* | ∆*upp* with a markerless deletion of *pedE* (5) | |
| ∆*pedE*∆*pedH* | ∆*upp* with a markerless deletion of *pedE and pedH* (5) | |
| ∆*pqq* | ∆*upp* with a markerless deletion of *pqqABCDE* (this study) | |
| *E. coli* BL21 (DE3) | *F^–^ ompT gal dcm lon hsdS_B_(r_B_^-^ m_B_^-^) λ(DE3 [lacI lacUV5-T7 gene 1 ind1 sam7 nin5])* | |
| *E. coli* DH5α | *fhuA2 lac(del)U169 phoA glnV44 Φ80' lacZ(del)M15 gyrA96 recA1 relA1 endA1 thi-1 hsdR17* | |
| **Plasmids** | | |
| pJeM1 | Rhamnose inducible production of EGFP (6) | |
| pJOE6261.2 | Suicide vector for markerless gene deletions (4) | |
| pMW43 | pJOE6261.2 based vector for the deletion of the PQQ synthesis gene cluster *pqqABCDE* (this study) | |
| pMW09 | pJeM1 based vector for rhamnose inducible expression of PedE with C-terminal 6x His-tag (this study) | |
| pMW10 | pJeM1 based vector for rhamnose inducible expression of PedH with C-terminal 6x His-tag (this study) | |
| pUC18-mini-Tn*7*T-Gm-lux | suicide vector for inserting single copies of *lux* transcriptional reporter fusions to the chromosome *via* a mini-Tn*7* element (2) | |
| pUC18-mini-Tn*7*T-*pedE*-*lux-Gm* | pUC18-mini-Tn*7*T-Gm-lux vector with the promoter of *pedE* driving transcription of *luxCDABE* (this study) | |
| pUC18-mini-Tn*7*T-*pedH*-*lux*-Gm | pUC18-mini-Tn*7*T-*lux*-Gm vector with the promoter of *pedH* driving transcription of *luxCDABE* (this study) | |
| pTNS2 | Helper plasmid for the integration of Tn7 into the chromosome (2) | |
| **Primer** | | |
| **Name** | **Sequence 5’ → 3’** | **Annealing** |
| MWH11 | CAATTCTTAAGAAGGAGATATACATATGACAATAAGATCGCTACCCGCCCTTTCC | 63 °C |
| MWH12 | GTCAATAAACCGGTAAGCTTAGTGGTGGTGGTGGTGGTGCTCGAGAGAGCCAGAGCCACGTTGTGCAGTCTTGTTGTCC | 63 °C |
| MWH13 | CAATTCTTAAGAAGGAGATATACATATGACCCGATCCCCACGTCG | 63 °C |
| MWH14 | AGTCAATAAACCGGTAAGCTTAGTGGTGGTGGTGGTGGTGCTCGAGAGAGCCAGAGCCTGGCTTGACGCTTGCCGTTTG | 63 °C |
| MWH34 | GCCGCTTTGGTCCCGGGCCTTGATGCTCAAGCC | 60 °C |
| MWH35 | GCGCTGAATGGAATTACTCCTTCCGAATGAGG | 60 °C |
| MWH36 | AGTAATTCCATTCAGCGCGGCCC | 65 °C |
| MWH37 | GCAGGTCGACTCTAGAGCGGCACCACCGGC | 65 °C |
| p2674-FSac | CGGGAGCTCGCAAGCAACACATTGCATTT | 60 °C |
| p2674-RPst | ACGCTGCAGGTGGTCAGGTGGTCATTGG | 60 °C |
| p2679-FSac | CGGGAGCTCAGTGTCAGCCACCTGTACCC | 60 °C |
| p2679-RPst | ACGCTGCAGTGCATCGTATTGCCACAGTT | 60 °C |

***Deletion of pqqABCDE gene cluster***

For the construction of the PQQ biosynthesis negative mutant ∆*pqq*, a previously described system for markerless gene deletion in *P. putida* KT2440 was used (4). In short, the integration vector pMW43 harboring the up- and downstream regions of the target gene cluster *pqqABCDE* was constructed and transformed into *P. putida* KT2440 *∆upp* (referred to as KT2440* within this manuscript). Kanamycin (Kan) resistant and 5-fluorouracil (5-FU) sensitive clones were selected on LB agar plates containing 40 µg ml^-1^ Kan and one of these was incubated in LB medium at 30°C for 24 h. The cell suspension was subsequently plated on M9 minimal agar plates containing 20 mM glucose and 20 µg ml^-1^ 5-FU. Clones that carried the desired gene deletion were identified by colony PCR of the 5-FU^r^ Kan^s^ clones using primer pair MWH34/MWH37.

***Expression and purification of PedE and PedH***

For production and purification of C-terminally His-tagged PedE and PedH, cells of *E. coli* BL21 (DE3) carrying plasmid pMW09 or pMW10 were grown in liquid LB medium supplemented with kanamycin and 1 mM CaCl_2_ at 37°C and 180 rpm. When cells reached an OD_600_ of ≥ 0.5 (Eppendorf, BioPhotometer) protein production was induced by addition of 0.2% [w/v] rhamnose and the medium was additionally supplemented with 0.6 µM pyrroloquinoline quinone (PQQ). Subsequently, cultures were shifted to 16°C and incubated at 180 rpm (HT Aerotron, Infors). After 16 h, cells were harvested by centrifugation (6000 ×g, 15 min, 4°C, Centrifuge 5810 R, Eppendorf), lysed by treatment with 1× Bugbuster solution (50 mM Tris-HCl pH 7.5 and 300 mM NaCl) supplemented with DNase (5 µg/ml) and lysozyme (5 µg/ml) and cell debris was removed by centrifugation (20000 ×g, 20 min, 4°C, Centrifuge 5424 R, Eppendorf). His-tagged PedE and PedH were purified from the cell-free extracts using affinity chromatography (His GraviTrap Talon® columns, GE Healthcare). For this 2 − 10 ml cell-free extract was loaded on a column, washed with 10 ml wash buffer (50 mM Tris-HCl pH 7.5, 300 mM NaCl, 5 mM imidazole) and eluted with 3 ml elution buffer (50 mM Tris-HCl pH 7.5, 300 mM NaCl, 150 mM Imidazole). Subsequently, excess ions were diluted 10 000-fold by dialysis with 50 mM Tris-HCl buffer pH 7.5. Protein purity was determined by visual inspection on SDS-PAGE (**Fig. S1**) and protein concentration was determined based on absorbance at 280 nm using a NanoDrop 2000 device. Eventually, enzymes were stored at -80°C as 1 mg ml^-1^ stock in 50 mM Tris HCl pH 7.5 containing 1 mg ml^-1^ bovine serum albumin (BSA) for stabilization.

***Amine source dependency of the enzymatic assay***

The optimal amine source and concentration was determined by using the enzyme activity assay described in materials and methods omitting imidazole in the presence of different amine sources. These included the commonly used amine sources ammonium chloride (45 mM) and ethylamine (5 mM) at reported concentrations as well as imidazole (6, 7). As imidazole gave an about 8-fold higher activity as ammonium chloride and an about 12-fold higher activity compared to ethylamine the ideal imidazole concentration was determined (**Fig. S5**). Therefore, the enzymatic assay was carried out at various imidazole concentrations and it was found that highest activities were measured for both PedE and PedH at concentrations above 25 mM (**Fig. S6**). For better visualization, the activities at varying imidazole concentrations were fitted by the least square method to a one site binding model based on the assumption that at v*_max_* 100% of the enzyme concentration is present associated with imidazole:

$$v_{0}=\frac{v_{max} \times[im]}{K_{D}+[im]}$$

Where [im] is the imidazole concentration, *v_0_* is the specific enzymatic activity at a certain imidazole concentration [im], *v_max_* is the maximal specific enzyme activity and *K_D_* is the dissociation constant.

***Phenazine methosulfate (PMS) dependency of the enzymatic assay***

The effect of the PMS concentration on the enzymatic assay was determined using 100 mM Tris HCl pH 8; 150 µM 2,6-dichlorophenol indophenol; 25 mM imidazole; 1 µM PrCl3; 1 µM PQQ; 12.5 µL substrate and 3 µg/ml PedH at various PMS concentrations. The highest activities are observed when using 500 µM or more PMS (**Fig. S7**).

***Pyrroloquinoline quinone (PQQ) affinity of the enzymes***

The optimal PQQ concentration for each enzyme was identified by using the enzymatic assay described in material and methods at varying PQQ concentrations. Highest activities were observed with 50 µM PQQ for PedE and with 0.5 and 1 µM PQQ for PedH (**Fig. S8**). For comparison purposes a dissociation constant *K_D_* was calculated by least-square analysis of the enzyme activities fitted with a one site binding model based on the assumption that at *v_max_* 100% of the enzyme concentration is present in PQQ bound form:

$$v_{0}=\frac{v_{max} \times[PQQ]}{K_{D}+[PQQ]}$$

Where [PQQ] is the PQQ concentration, *v_0_* is the specific enzymatic activity at a certain PQQ concentration [PQQ], *v_max_* is the maximal specific enzyme activity and *K_D_* is the dissociation constant.

***Metal binding affinity of the enzymes***

To test the metal binding affinities of PedE and PedH, a similar set-up as described in material and methods was used omitting CaCl_2_ for PedE or PrCl_3_ for PedH in the assay solution. Various concentrations of different metals were added prior to incubation at 30°C. These included LaCl3, CeCl_3_, PrCl_3_ and CaCl_2_. To allow comparison of different enzymes and metals, a dissociation constant *K_D_* was calculated by least-square analysis of the enzyme activities fitted with a one site binding model based on the assumption that at *v_max_* 100% of the enzyme concentration is present in metal bound form:

$$v_{0}=\frac{v_{max} \times[M]}{K_{D}+[M]}$$

Where [M] is the metal concentration, *v_0_* is the specific enzymatic activity at a certain metal concentration [M], *v_max_* is the maximal specific enzyme activity with a given metal *M* and *K_D_* is the dissociation constant.

**References**

1. **Gibson DG, Young L, Chuang R-Y, Venter JC, Hutchison C a, Smith HO.** 2009. Enzymatic assembly of DNA molecules up to several hundred kilobases. Nat Methods **6**:343–345 doi:10.1038/nmeth.1318.

2. **Choi K-H, Gaynor JB, White KG, Lopez C, Bosio CM, Karkhoff-Schweizer RR, Schweizer HP.** 2005. A Tn7-based broad-range bacterial cloning and expression system. Nat Methods **2**:443–448 doi:10.1038/nmeth765.

3. **Choi K-H, Kumar A, Schweizer HP.** 2006. A 10-min method for preparation of highly electrocompetent *Pseudomonas aeruginosa* cells: Application for DNA fragment transfer between chromosomes and plasmid transformation. J Microbiol Methods **64**:391–397 doi:10.1016/j.mimet.2005.06.001.

4. **Graf N, Altenbuchner J.** 2011. Development of a method for markerless gene deletion in *Pseudomonas putida*. Appl Environ Microbiol **77**:5549–5552 doi:10.1128/AEM.05055-11.

5. **Mückschel B, Simon O, Klebensberger J, Graf N, Rosche B, Altenbuchner J, Pfannstiel J, Huber A, Hauer B.** 2012. Ethylene glycol metabolism by *Pseudomonas putida*. Appl Environ Microbiol **78**:8531–9 doi:10.1128/AEM.02062-12.

6. **Jeske M, Altenbuchner J.** 2010. The *Escherichia coli* rhamnose promoter rhaP BAD is in *Pseudomonas putida* KT2440 independent of Crp–cAMP activation. Appl Microbiol Biotechnol **85**:1923–1933 doi:10.1007/s00253-009-2245-8.

7. **Chattopadhyay A, Förster-Fromme K, Jendrossek D.** 2010. PQQ-dependent alcohol dehydrogenase (QEDH) of *Pseudomonas aeruginosa* is involved in catabolism of acyclic terpenes. J Basic Microbiol **50**:119–24 doi:10.1002/jobm.200900178.

8. **Anthony C, Zatman LJ.** 1964. The microbial oxidation of methanol. 2. The methanol-oxidizing enzyme of Pseudomonas sp. M 27. Biochem J **92**:614–21.
